# Citrus genomes: past, present and future

**DOI:** 10.1093/hr/uhaf033

**Published:** 2025-02-04

**Authors:** Upuli Nakandala, Agnelo Furtado, Robert J Henry

**Affiliations:** Queensland Alliance for Agriculture and Food Innovation, University of Queensland, Sir Fred Schonell Drive, St Lucia, Brisbane 4072, Australia; ARC Centre of Excellence for Plant Success in Nature and Agriculture, University of Queensland, Sir Fred Schonell Drive, St Lucia, Brisbane 4072, Australia; Queensland Alliance for Agriculture and Food Innovation, University of Queensland, Sir Fred Schonell Drive, St Lucia, Brisbane 4072, Australia; ARC Centre of Excellence for Plant Success in Nature and Agriculture, University of Queensland, Sir Fred Schonell Drive, St Lucia, Brisbane 4072, Australia; Queensland Alliance for Agriculture and Food Innovation, University of Queensland, Sir Fred Schonell Drive, St Lucia, Brisbane 4072, Australia; ARC Centre of Excellence for Plant Success in Nature and Agriculture, University of Queensland, Sir Fred Schonell Drive, St Lucia, Brisbane 4072, Australia

## Abstract

Over the past decade, genome sequencing and assembly approaches have been greatly improved, resulting in the assembly of many genomes for citrus, including wild, domesticated, and citrus-related genomes. Improvements in technologies have led to assembled genomes with higher completeness, contiguity, quality, and accuracy that have greatly facilitated annotation and analysis. This review summarizes the evolution of the sequencing, assembly, and annotation technologies leading to citrus genomes over the past 11 years, a comprehensive evaluation of their quality, contiguity, and completeness, and the major findings and applications. Of the 50 genomes now available, 35 have been assembled to chromosome level and 15 to draft level, and 14 were haplotype-resolved assemblies. To date there have been four pangenome-wide studies for citrus. The very recent genomes assembled with long-read sequencing have achieved >99% and >98% assembly and annotation completeness (BUSCO), respectively. However, some early genomes are not of the same high quality as more recently sequenced genomes and would benefit from re-sequencing. A more comprehensive pangenome based upon a larger set of species and genotypes assembled at the haplotype level would allow genomics to deliver the maximum benefits for citrus improvement and research.

## Introduction

Sequencing technologies have been developed over the past few decades in two main phases: first-generation (low-throughput Sanger technology) and next-generation sequencing (NGS) technology involving high throughput due to massive or massively parallel sequencing approaches. NGS technologies include second-generation technology involving short-read sequences (Illumina) and third-generation, long-read technologies [PacBio and Oxford nanopore (ONT)] [1]. Sanger sequencing was limited in its application, particularly in genome assembly, due to its low throughput, high cost, and labor intensity. NGS involving second-generation sequencing has facilitated the sequencing of relatively smaller genomes with high throughput, high accuracy, and low cost [1]. However, the high heterozygosity, high repeat content, complexity, and polyploidy of many plant genomes have challenged the generation of complete and accurate assemblies with second-generation NGS sequencing due to their short read lengths [2]. Third-generation NGS sequencing techniques with longer read lengths (average length of >10 kb) and high accuracy have been more successful than the previous techniques in achieving contiguous genomes and accurate characterization of the genomic features [3].

Citrus is an important fruit crop, produced in more than 140 countries around the world [4]. Currently, there are 50 citrus genome sequences published and publicly available since 2013. Among them, there are four pangenome-wide studies, some based upon completely *de novo* assembled genomes [5], while others employed reference genomes together with *de novo* assembly in their analysis [6–8]. There is only one report summarizing the genomes of a few citrus varieties, which was available before 2017 [9]. These genomes were assembled using Sanger, Illumina, and PacBio RSII. Since that time, PacBio CLR (continuous long reads), PacBio CCS (circular consensus sequencing) or HiFi sequencing, ONT sequencing, Illumina sequencing, and Hi-C chromatin mapping approaches were widely adopted in assemblies, and 42 additional genomes have been reported to date. This review summarizes research leading to the 50 citrus genome assemblies that have been reported, and discusses their completeness, contiguity, annotation, phasing, important gene families, and evolutionary events, and the transcriptomic and epigenomic studies that were based on these genomes. Moreover, the applications of genomes in gene mining and breeding practices, and population genetics, and a comparison with other horticultural species to understand the unique features of citrus genomics research have been provided. This will provide a valuable and comprehensive resource collating current knowledge on citrus genomes and existing challenges and identifying future implications for research on citrus.

## Sequencing technologies used for citrus genome assemblies

The very first genomes available for citrus in 2013 were for *Citrus sinensis* based upon Illumina sequencing [10], and in 2014 for *C. clementina* based on Sanger sequencing [11]. To avoid the complexities of assembling outbred diploid genomes from short read sequences, these genome projects used haploid and dihaploid materials. In the following years, Illumina sequencing was used to sequence some other wild and cultivated citrus species. Some of the assembled genomes indicating the evolution of the sequencing platforms are given in Table 1 and more extensive information on all the assembled genomes to date are given in Supplementary Data Table S1. With the advent of third-generation sequencing techniques, the genome projects used a combination of long-read and short-read sequencing data such as PacBio RSII, PacBio CLR, PacBio HiFi, ONT, and Illumina for genome assemblies (Table 1, Supplementary Data Table S1). HiFi sequencing (involving the repeated sequencing of the same molecule) has subsequently greatly improved the quality of sequencing data and the resulting genome assemblies [12]. Chromatin mapping using Hi-C data has recently been used for developing more complete, fully haplotype-resolved, chromosome-level genome assemblies of some cultivated varieties, including *C. sinensis* [7, 13], *C. limon* [14], and *C. changshanensis* [15], and some wild citrus species, including *C. australasica* [16, 17], *C. inodora*, and *C. glauca* [17, 18]. Moreover, optical mapping was used for further improvements in some genome assemblies [6], but has been used less with the arrival of improvements in sequencing technology such as HiFi and the use of Hi-C.

**Table 1:** Sequencing and assembly approaches and outcomes for some available genomes of domesticated and wild citrus species and citrus relatives.

**Common name**	**Scientific name**	**Sequencing platforms, data yield (Gb) and coverage (X), and mapping techniques**	**Genome assembly tools**	**Estimated genome size (Mb)**	**Assembly contiguity (N50) (Mb) and completeness (BUSCO) and CEGMA (%)**	**Haplotypes resolved (HR)/haplotypes not resolved (HNR), telomeres reported (TR)/telomeres not reported (TNR)**	**Reference**
Valencia sweet orange	*C. sinensis* cv. ‘Valencia’	Illumina (214X), genetic maps	SOAPdenovo and Opera	367.0	Scaffold N50: 1.69	HNR, TNR	[10]
Valencia sweet orange (DVS)	*C. sinensis* cv. ‘Valencia’	PacBio CLR (143.9, 420X)	MECAT2, Canu, Falcon		Contig N50: 15.4 Assembly BUSCO: 98.7%, QV: 50.6	HR, TNR	[19]
Navel orange	*C. sinensis* Osbeck cv. ‘Gannanzao’	PacBio HiFi, Hi-C	Hifiasm, Lachesis	334.7	Scaffold N50: 31.86 Assembly BUSCO: 94.6 CEGMA score: 93.55	HNR, TNR	[20]
Minong sweet orange	*C. sinensis* cv. ‘Minong’	PacBio HiFi (25.2, 80X), ONT (29.2), Hi-C (100X)	Hifiasm, RagTag, 3D-DNA		Contig N50: 20.6, 16.4 Assembly BUSCO: >98.5	HR, TNR	[21]
Clementine mandarin	*C. clementina*	Sanger (7X), genetic maps	Arachne	301.4		HNR, TNR	[11]
Citrumelo	*Swingle citrumelo*	Illumina (200X)	CLC genomic workbench v6.0.1	380.0	Scaffold N50: 11.4 kb CEGMA score: 96.3	HNR, TNR	[22]
Pummelo	*C. maxima/C. grandis*	Illumina, PacBio CLR, Hi-C	Canu, Minimap2, purge_dups, NextPolish, ALLHiC, MUMMER4	349.0	Contig N50: 1.74 Assembly BUSCO: 99.1%	HNR, TNR	[23]
Satsuma mandarin	*C. unshiu* Marc.	Illumina and PacBio RSII	PLATANUS, Opera and PBJelly	359.7	Scaffold N50: 0.39 Assembly BUSCO: 94.2% Annotation BUSCO: 92.1%	HNR, TNR	[24]
Tangshan wild mandarin	*C. reticulata*	Illumina (64.2, 199.7X)	PLATANUS and GapCloser software		Scaffold N50: 1.7 Assembly BUSCO: 96%	HNR, TNR	[25]
Hongkong kumquat	*Fortunella hindsii*	PacBio CLR (57.5, 145X) and Illumina (51.3), 10X genomic data (43.2)	MECAT, ARCS and LINKS	374.0	Scaffold N50: 5.2 Assembly BUSCO: 95.1%	HNR, TNR	[26]
Trifoliate orange	*Poncirus trifoliata*	PacBio CLR (140X), Illumina (80X), Hi-C	Falcon, HiRise, Juicebox	264.9	Scaffold N50: 27.7 Assembly BUSCO: 97.2%	HNR, TNR	[27]
	*P. polyandra*	ONT, Hi-C	NextDenovo, Racon, NextPolish, Lachesis	315.8	Contig N50: 7.57 Scaffold N50: 32.07 Assembly BUSCO: 98.82%	HNR, TNR	[28]
Lemon	*C. limon*	PacBio CCS (21.9), ONT [23], Hi-C [40]	Hifiasm, Verkko, ALLHiC, TGS-GapCloser		Contig N50: 35.6 Assembly BUSCO: 98.7% QV of individual chromosomes: 62–81	HR, TR	[14]
Australian round lime	*C. australis*	PacBio CCS (58.5, 172X), Hi-C (99.1292X)	Hifiasm, SALSA	328.5 (collapsed), 325.8 (hap1), 300 (hap2)	Scaffold N50: 30.7–35.1 BUSCO: 97.4%–98.8%	HR, TR	[12]
Australian finger lime	*C. australasica* cv. ‘Rainbow’	PacBio CCS (75.9, 223X), Hi-C (116, 341X)	Hifiasm, BWA + Arima mapping + SALSA	344.2 (collapsed), 321.1 (hap1), 323.2 (hap2)	Scaffold N50: 32.3–35 BUSCO: 98.9%–99.1%	HR, TR	[16]
Russell River lime	*C. inodora*	PacBio CCS, Hi-C	Hifiasm, Falcon-Phase, Phase Genomics’ Proximo Hi-C genome scaffolding platform	303.7 (primary) 298.8 (alternate)	Scaffold N50: 28.9 BUSCO: 96.68%–96.70%	HR, TNR	[17]
Dessert lime	*C. glauca*	PacBio CCS (61, 180X), Hi-C (90.4, 266X)	Hifiasm, BWA + Arima mapping + SALSA	340.2 (collapsed), 318 (hap1), 311.6 (hap2)	Scaffold N50: 32.4–33.5 BUSCO: 98.4%–99.5%	HR, TR	[18]
Mount white lime	*C. garrawayi*	PacBio CCS (64.3, 189X), Hi-C (96.2283X)	Hifiasm, BWA + Arima mapping + SALSA	316.8 (collapsed), 286.8 (hap1), 312.7 (hap2)	Scaffold N50: 28.9–30 BUSCO: 98.3%–98.6%	HR, TR	[18]
Huyou	*C. changshanensis*	PacBio HiFi (25, 84X), Hi-C (64, 212X), Illumina (73X)	Hifiasm, 3D-DNA	354.6	Contig N50: 30.1–32.4 Assembly BUSCO: 98%–98.2% QV: 42.74–43.26 LAI: 15.4–19.54	HR, TNR	[15]

## The status of current genome assemblies

### Assembly approaches

The early genome assembly approaches used a range of different software tools to assemble the sequencing reads into contigs and scaffolds, which were then integrated with genetic maps to be developed to chromosome level. The contig assembly, scaffolding, and gap filling of the very first citrus genome of *C. sinensis* was performed using Illumina data and the SOAPdenovo assembler, and was further improved using the scaffolder Opera (Supplementary Data Table S1). The genome of *C. clementina* was based upon Sanger sequence data and an Arachne whole-genome shotgun assembler (Supplementary Data Table S1). The scaffolds of these assemblies were anchored to a genetic map to generate pseudochromosomes. The scaffolds of five later genomes were also anchored to genetic maps by molecular markers to construct the pseudochromosomes [6, 24, 29, 30], and the scaffolding of other genomes was performed using Hi-C data (Supplementary Data Table S1). The numbering and orientation of the pseudochromosomes of the genome assemblies were based on either the *C. clementina* genome [11] or the *C. sinensis* genomes (Supplementary Data Table S1). The pseudochromosome construction for one of the *C. limon* genomes [31] and Australian limes was also assisted by reference genomes [16, 18, 32].

The assembly of *Swingle citrumelo* was done using Illumina reads in the CLC genomic workbench, while another four draft genomes of *C. grandis*/*C. maxima*, *C. medica*, *C. ichangensis*, and *Atalantia buxifolia* were produced using the SOAPdenovo package [29]. Later on, two other genomes for *C. grandis*/*C. maxima* were developed in two consecutive years: 2022 and 2023. One genome was developed using NECAT, Racon, NextPolish , Juicer, and 3D-DNA software utilizing ONT and Hi-C sequencing data. The other genome was developed using Canu, Minimap2, purge_dups, NextPolish , ALLHiC, and MUMMER4 tools by employing Illumina, PacBio CLR, and Hi-C sequencing data. In 2017, the genome of *C. unshiu* was released based upon contig and scaffold assemblies generated using PacBio RSII and Illumina data using the assembler PLATANUS, optimization of scaffolds was done using Opera, and gap-closing was performed using PBJelly (Supplementary Data Table S1).

The genome of *C. reticulata* was first assembled using Illumina short reads with the help of PLATANUS assembler in 2019, and the gaps were filled with GapCloser software. Recently, another genome was made available for *C. reticulata* using ONT and MGI short reads, assembled in NextDenovo. The first genome of *Fortunella hindsii* was produced using MECAT, ARCS, and LINKS software by employing PacBio CLR and Illumina sequencing in 2019. Later on, another genome for *F. hindsii* sequenced by PacBio CLR, Illumina, and Hi-C, and assembled using Canu, smartdenovo, and Lachesis assemblers became available in 2022 (Supplementary Data Table S1). There are three genomes available for *Poncirus t*o date. Two of them were for *P. trifoliata* and one was for *P. polyandra*. Among the two genomes of *P. trifoliata*, one became available in 2020, assembled with PacBio CLR, Illumina, and Hi-C data with the help of Falcon, HiRise, and Juicebox software, while the other was released in 2021 employing PacBio RSII and Illumina data with the help of Falcon/Falcon_Unzip, Quiver, and Pilon software. The *P. polyandra* genome was developed with NextDenovo, Racon, NextPolish, and Lachesis assemblers using ONT and Hi-C data.

The first genome for *C. limon* became available in 2021, assembled with ONT and Illumina reads using MaSuRCA and Falcon software, and this was one of the first phased genomes available for citrus (Supplementary Data Table S1). A more advanced genome was then available in 2023 using an integrated sequencing approach involving PacBio CCS, ONT, and Hi-C reads. The sequencing reads were assembled using a combination of software including Hifiasm, Verkko, ALLHiC, and TGS-GapCloser, creating a gap-free, more complete, and haplotype-resolved genome for lemon. Another recent genome was released for *C. limon* in 2024, assembled using PacBio CLR, Illumina, and Hi-C technologies (Supplementary Data Table S1).

With the advent of third-generation sequencing, several genomes have been produced for *C. sinensis* since 2021. The first haploid genome sequenced by third-generation techniques was assembled using a combination of software (HGAP, quiver and pilon, SSPACE, GapCloser). The first phased genome for *C. sinensis* was then available in 2023, sequenced using PacBio CLR, and assembled using MECAT2, Canu, and Falcon tools. Another unphased genome that was assembled using Hifiasm and Lachesis was also publicly available in 2023. More recently, another two phased diploid genomes were developed for *C. sinensis*. One was developed in 2024 using Hifiasm, RagTag, and 3D-DNA and the other was developed in 2023 using Hifiasm, ALLHIC, and Juicebox software (Supplementary Data Table S1). Several genomes, including six citrus species and six citrus-related species, were generated in one study for the first time using PacBio, ONT, and Hi-C sequencing. A combination of software including SMRTdenovo, miniasm, NextDenovo, NECAT, Canu, Racon, and NextPolish was used for these genome assemblies and 3D-DNA was used for their scaffolding (Supplementary Data Table S1).

The genomes of Australian wild limes also recently became available with more recent sequencing technologies. Among them, the genome of *C. australasica* has been assembled by several groups due to its importance among the native limes. One high-quality and fully haplotype-resolved genome was developed by PacBio HiFi and Hi-C sequencing reads that were assembled by Hifiasm and BWA + Arima mapping + SALSA tools [16]. Another phased genome was developed with the same sequencing technologies, and assembled using Hifiasm, Falcon-Phase, and Phase Genomics’ Proximo Hi-C genome scaffolding platform [17]. The latest genome for *C. australasica* was a haploid genome sequenced by ONT reads and assembled using NextDenovo, NextPolish, and RagTag software [32]. In addition, the genomes of other Australian limes, such as *C. inodora*, *C. glauca*, *C. australis*, and *C. garrawayi*, have also been assembled using very recent genome sequencing and assembly approaches [12, 18] (Supplementary Data Table S1).

Additionally, the genome of *C. changshanensis* has been produced using PacBio HiFi, Illumina, and Hi-C data with Hifiasm, and 3D-DNA software [15] (Supplementary Data Table S1). Three genomes were recently developed with Illumina, ONT data, and Bionano optical mapping using the NECAT and HAPO-G software tools.

### Genome annotation

Genome annotation encompasses sequence-repeat annotation and gene prediction. Since the beginning of citrus genome assembly, many tools have been using for sequence-repeat detection and gene prediction. The main types of sequence-repeat elements in citrus genomes are LTR elements, unclassified repeats, DNA transposons and interspersed repeats (LINEs and SINEs). The total sequence-repeat content of citrus genomes has been reported to range from 20% to 61% (Supplementary Data Table S2). The gene models in these genomes were annotated using different *ab initio* gene prediction tools, homology support (EST or/and protein databases) and RNA-seq evidence (Supplementary Data Table S2). The total number of gene models annotated in citrus genomes has ranged from 20 815 to 41 852; some gene models in some genomes could not be localized within chromosomes and were therefore annotated in unplaced scaffolds (Supplementary Data Table S2). The total repeat and gene contents of the annotated genomes, which are measures of completeness of an assembly and annotation, depend upon the accuracy and advancement of sequencing technologies, assembly, and annotation software. Therefore, the very early genomes developed with Sanger and next-generation sequencing are not as complete as recent genomes developed with third-generation sequencing and more sophisticated assembly and annotation tools.

### Improvement of assemblies and associated novel discoveries as a consequence of advances in sequencing and assembly technologies

#### Assembly quality, contiguity (N50), and completeness (total BUSCO/CEGMA/LTI, gaps, and telomeres)

**Figure 1:**
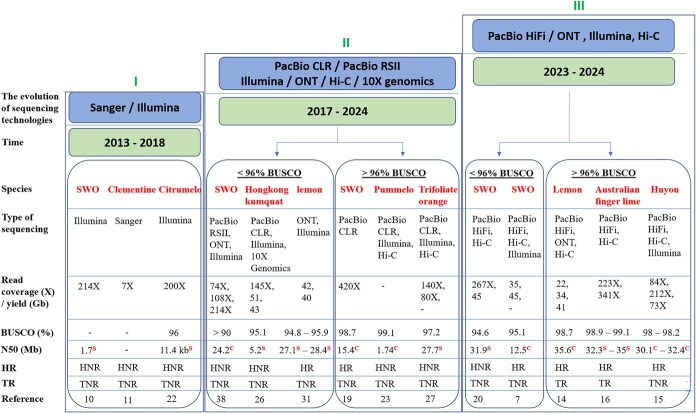
Changes in the quality, contiguity, and completeness of a few genome assemblies over time with advancements of sequencing technologies. The sequencing approaches used in previous studies can be categorized into three phases. The first phase used Sanger or Illumina sequencing, generating draft assemblies from 2013 to 2018. Some studies did not report their completeness and contiguity. Other genomes had very low BUSCO levels and contiguities (N50). None of the genomes were haplotype-resolved and no telomeres were found in them. In the second phase, third-generation sequencing technologies such as PacBio RSII and PacBio CLR with other sequencing technologies such as Illumina, 10X genomics, and Hi-C were adopted between 2017 and 2024. These genomes can be categorized into two types based on their completeness in terms of BUSCO scores. Their contiguities have greatly improved compared with the previous phase of sequencing technologies and two were phased into haplotypes. In the third phase, in addition to the ONT, Illumina, and Hi-C sequencing technologies, PacBio HiFi was adopted in assemblies. This has greatly revolutionized the genomes, with very high contiguities at the contigs and scaffold levels. Some of the genomes had <96% total BUSCO values and they were not haplotype-resolved. Many genomes having a greater BUSCO value (>98%) were phased into haplotypes and telomeric regions were identified in these genomes, giving rise to more complete assemblies. SWO, *C. sinensis*. Clementine, *C. clementina*; Citrumelo, *Swingle citrumelo*; Hongkong kumquat, *Fortunella hindsii*; lemon, *C. limon*; Pummelo, *C. maxima*/*C. grandis*; Trifoliate orange, *Poncirus trifoliata*; Australian finger lime, *C. australasica*; Huyou, *C. changshanensis*; HR, haplotypes resolved; HNR, haplotypes not resolved; TR, telomeres reported; TNR, telomeres not reported.

The genomes sequenced by third-generation sequencing techniques are of high quality, high contiguity and high completeness compared with those sequenced by early sequencing technologies. The use of more advanced contig and scaffolding assembly approaches has also largely improved the citrus genomes. Changes in the quality and completeness of the assemblies over time with the evolution of sequencing technologies are depicted in Fig. 1 using a selected set of genomes. There was no indication of assembly contiguity and completeness for some early genomes sequenced by Sanger/Illumina sequencing platforms. Early genomes sequenced using PacBio RSII, CLR, or ONT with Illumina, Hi-C, and 10X genomics were found to have improved contiguities compared with Sanger or Illumina sequencing alone (Fig. 1, Supplementary Data Table S1). The BUSCO value, a measure of genome assembly completeness, has been lower than 96% for most of the genomes sequenced by first-generation sequencing techniques; however, it was mostly greater than 98% (98%–99% or >99%) for genomes sequenced by next-generation sequencing (Supplementary Data Table S1).

Some genomes categorized in phase II in this study (Fig. 1) had lower BUSCO scores (<96%), while some had higher BUSCO scores (>96%). Some genomes with a high coverage of PacBio CLR data (420X) were shown to have an increased assembly BUSCO score (98.7%) [19], whereas other genomes with reasonably high coverages of PacBio (395.2X), Illumina (140X), and Hi-C data (128X) were found to have a relatively low BUSCO score (95%) [33]. However, the latter genome was found to have a high CEGMA (Core Eukaryotic Genes Mapping Approach) score. Different BUSCO scores in different genomes might possibly be due to different lineages that were used in BUSCO analysis (viridiplantae or eukaryota), different coverage of sequencing reads used, and different assembly approaches used. Some genomes with high assembly BUSCO scores (99.1%) have used a combination of sequencing data (PacBio CLR, Illumina, and Hi-C) with no coverage details [23].

In recent genomes sequenced by PacBio CCS (HiFi) sequencing with integration of other data, such as Hi-C, ONT, Illumina, or optical mapping, contiguity at the contig level (contig N50) has been increased (now >10 Mb), and that of the scaffolding level (scaffold N50) has also become greater than 25 Mb due to the assembly of large chromosome-scale scaffolds (Fig. 1, Supplementary Data Table S1). Some of these genomes had <96% total BUSCO scores and others had >96% BUSCO scores. The genomes having lower coverages of PacBio HiFi and Hi-C data [7] were found to have lower BUSCO scores (<96%). Also, those having a higher coverage of PacBio HiFi data but a lower coverage of Hi-C data [20] were also found to have a relatively low BUSCO score (<96%). In contrast, the genomes having high coverages of both PacBio HiFi and Hi-C data [12, 15, 16, 18] and the genomes assembled with additional data such as ONT reads in addition to PacBio HiFi and Hi-C reads [14, 21] were found to have increased BUSCO scores (>96%).

Other measures for assembly completeness, such as LTR assembly index (LAI), CEGMA score, and QV, have been used in a few genomes to measure gene completeness and the quality of genome assemblies. The score of LAI for one of the *C. australasica* genomes was 15.19, and those of *C. changshanensis* assemblies were ranged between 15.4 and 19.54 (Supplementary Data Table S1). Based on these scores, the genomes could be considered to be of reference quality (10 ≤ LAI < 20) and were categorized into neither draft (0 ≤ LAI < 10) nor gold qualities (20 ≤ LAI) [34]. Three genomes have been evaluated with CEGMA score, which was 93.55 for *C. sinensis* Osbeck cv. ‘Gannanzao’, 96.3 for *Swingle citrumelo* and 97.18 for *C. limon* (Supplementary Data Table S1). The CEGMA score is based on a set of reliable genes conserved in eukaryotes and higher values indicate more complete gene annotations [35]. The QV value assessed by Merqury is an indication of the assembly quality and it is based on *k*-mers [36]. Two genomes were found to have sufficiently good QV scores (*C. sinensis* cv. ‘Valencia’, 50.6; *C. changshanensis*, 42.74–43.26), while one of the *C. limon* genomes was found to have high QV scores for individual chromosomes [62–81]. The most recent genome of *C. australasica* had a relatively low QV score, which was 27.68. These additional measures are important in evaluating the completeness and quality of genomes; however, they have not been used in many genomes.

The presences of gaps and telomeres in genome assemblies are other measures of completeness in a genome. Attempts were made to fill the gaps in early genomes with short paired-end reads [22, 24, 25, 29] or mate-paired reads [24, 29], and those in later genomes were closed with long reads such as PacBio CLR and ultra-long ONT [24, 37], and PacBio HiFi and ultra-long ONT [14]. However, gaps still remain in some genomes. To date, only the genomes of *C. reticulata* [38] and *C. limon* [14] have been declared to be gap-free, in which each chromosome was represented by a single contig. Although the *C. reticulata* genome was gap-free, only three chromosomes were assembled with telomeres at both ends, and four were assembled with telomeres at one end, indicating the chromosomes were not fully complete [38]. In the *C. limon* genome also, the telomeres were not found on all the chromosomes in haplotype A, indicating incompleteness. Among the Australian wild limes, *C. australis*, *C. australasica*, *C. garrawayi*, and *C. glauca* had four, four, four, and seven complete chromosomes with telomeres at both ends, respectively. However, none of the chromosomes of *C. inodora* were telomere–telomere (T2T) [12, 16, 18]. Only the recently available genomes in 2023 and 2024 sequenced by PacBio HiFi reads have been reported as having telomeres (Fig. 1). The corresponding locations of the centromeres have been deduced in *C. reticulata*, *C. medica*, and *C. micrantha* and were identified with reduced gene densities and high repeat contents [6].

#### Development of haplotype-resolved assemblies

The development of haplotype assemblies was initiated in 2021 for *C. limon* with the advent of third-generation sequencing [31]. Since then, different studies have used different approaches to phase genomes. All the other haplotype assemblies became available in 2023 and 2024 (Fig. 1). Some studies have used *de novo* methods for phasing, whereas others have used reference-guided methods (aligning contigs against parental genomes). Some approaches have also used different pipelines to correct subsequent switching errors in the phased haplotypes [15, 17]. The phasing of the genome of ‘Valencia’ sweet orange was performed by utilizing the high intra-genomic heterozygosity, which has created a high allelic variation between the haplotypes [19]. The genome was phased by mapping the contigs of the Canu assembly against the remaining contigs of the assembly to detect the orthologous regions, followed by using pb-falcon to phase the unphased collapsed regions [19]. Another approach used the HiFi reads and Hi-C reads integration option in Hifiasm to develop haplotype-resolved assemblies [12–14, 16, 18]. The genome of *C. limon* was phased by mapping the contigs against the parental genomes along with the purge haplotig pipeline [31].

To date, there are 14 genome assemblies for *C. sinensis*, *C. limon*, Australian wild limes, and *C. changshanensis* that are phased. Among them, three phased genomes are available for *C. sinensis* [7, 13, 19], two are available for *C. limon* [14, 31], *C. australasica* [16, 17], *C. inodora* [17, 18], and *C. glauca* [17, 18], and one is available for *C. changshanensis* [15], *C. australis* [12], and *C. garrawayi* [18]. Of the two phased assemblies of most of the genomes, a major portion shared a high nucleotide similarity. Most of the genes were found to be syntenic (same genes present) [12, 15, 17, 18] or collinear (same genes present in the same order) [18, 19] between the homologous chromosomes. On the other hand, the Newhall navel orange genome published in 2023 [7] identified a relatively low similarity between the homologous chromosomes of the two haplotypes.

The haplotype assemblies are different from each other in terms of their size and the total gene number (Supplementary Data Table S3). The size difference of the nine chromosomes between the two haplotypes was the highest in one of the *C. limon* genomes (34.2 Mb) [14] and was the lowest in *C. garrawayi* (1.3 Mb) [18]. The difference in the total number of genes between haplotypes within the nine chromosomes was highest in one of the *C. australasica* genomes (4089) [16], while it was lowest in *C. australis* (69) [12] (Supplementary Data Table S3). In addition, the number of repeat elements was also found to be different between the haplotypes.

Apart from the syntenic regions, structural variants, including single-nucleotide variants, small indels, insertions, deletions, tandem duplications, translocations, inversions, and highly diverged regions, were also found between the two haplotypes (Supplementary Data Table S3).

Haplotype-resolved assemblies provide important information related to their parental origins, structural variations and similarities, and haplotype-specific alleles, which broaden our understanding about their evolution and facilitate their use in breeding. The phased assemblies of sweet orange have been used to determine the parental origins of the homologous chromosomes [13, 19]. As a result, the regions of whole haplotype assemblies and individual chromosomes derived from mandarin and pummelo were determined. The results suggested that sweet orange was more likely derived from a maternal parent, which is a complex hybrid with a larger proportion from pummelo and a smaller proportion from mandarin, and from a paternal parent that is a mandarin introgressed with a small portion of pummelo in its nuclear genome [19]. Similarly, the parental origins of lemon have been characterized using a phased lemon genome [14]. Accordingly, a major portion (53.5%) was found to be derived from citron and the rest of the genome was found to be derived from mandarin and pummelo (~23%).

Phasing moreover enabled the detection of haplotype-specific genes in each assembly [13, 19]. These hemizygous genes, where only one allele was present in one haplotype, were profoundly enriched in certain biological processes, such as defense response, hormone signaling, and sexual reproduction [19]. Certain biallelic genes with heterozygous variations (structural variants and SNPs) were found to have allelic expression differences during fruit development [13]. Interestingly, a positive, significant correlation was found between the heterozygous allelic variations and the enrichment of the genes with allele-specific expressions in *C. sinensis*. Some alleles with transposable elements (TEs) inserted within the promoter region were highly expressed, while other alleles with no TE insertion were weakly expressed, indicating allele-specific expression patterns for some genes regulating fruit development [13]. This study further identified more somatic mutations in haplotype-resolved assemblies compared with single collapsed genomes, revealing the importance of phasing.

#### Development of pangenomes

To date, three pangenome-scale studies have been performed in citrus. A pangenome represents the complete set of non-redundant DNA sequences in a species or a population [39]. Citrus pangenomes have been constructed based upon structural variants [5] and gene families [7]. A study with 18 citrus and citrus-related genomes revealed 34 564 gene families, of which 35.8% were core and softcore gene families, 55.4% were dispensable gene families, and 8.8% were special gene families [5]. This study revealed South Central China as the origin of the genus *Citrus* and the ancient Indian Plate as the origin of *Citrus*-related genera. This study further demonstrated the sequence and expression variations of the *PH4* gene resulting in an increase of citric acid in these genomes. Another study included 10 genomes of wild, primitive, cultivated *Citrus* and related genera that were tolerant and susceptible to huanglongbing (HLB) disease, and revealed more unique immunity-related genes in HLB-susceptible accessions [7].

Another study of five *Citrus* and related genera analyzed the cyclic nucleotide-gated channels (*CNGC*s) gene family revealing structural alterations and expansions in genomes and high expression in response to drought [8]. Another pangenome comprising four ancestral cultivated varieties—*C. reticulata*, *C. medica*, *C. micrantha*, and *C. maxima*—revealed SNP variants differentiating the four varieties. This study revealed that many cultivated mandarins have a genome largely derived from *C. reticulata* and some have introgressions from *C. maxima*. In cultivated pummelos, the genome is primarily constituted of *C. maxima*, while some varieties were found to have *C. reticulata* and *C. medica* introgressions. Most citrons were pure representatives of *C. medica*, while a very few had introgressions from *C. micrantha*. Many other cultivars displayed different genomic admixture patterns from the four ancestral cultivars. The pangenome identified the critical biological processes shared among the four cultivars and unique genes corresponding to their adaptive traits.

## Important gene families annotated in genomes

### Immunity-related genes

The genome of *C. limon* has unveiled expanded genes that may play crucial roles against HLB. These genes belong to plant–pathogen interactions, hormone signaling, MAPK signaling, and other genes conferring phloem-based defense and associated with cell wall synthesis, degradation, and modification [14]. The genomes of Australian wild limes, including *C. australis*, *C. australasica*, and *C. glauca*, were annotated with many defense genes encoding stable antimicrobial peptides (SAMPs), guanine nucleotide-binding proteins, pathogenesis-related proteins, leucine rich repeats (LRRs), cysteine-rich receptor-like protein kinases (CRKs), and other genes that may be responsible for their HLB tolerance. The disease resistance genes (R genes) in Australian limes were classified into different categories based on their domains [17]. Among the Australian limes, the highest number of genes for terpene biosynthesis were identified in *C. australasica* [18]. In contrast, a comparatively high number of receptor-like proteins (RLPs) and disease resistance protein (RPS2) were annotated in the *C. australis* genome. The number of other immunity-related genes, such as thiamine metabolism and glutathione metabolism were found to be the highest in *C. glauca*. These findings revealed the potential involvements of these genes in resistance against HLB in Australian limes.

Another study related to HLB has found several immunity-related genes encoding proteins such as NBS (nucleotide binding site)-LRR, PLCP (cysteine protease-like protein), lectin, leucine-rich repeat receptor-like protein kinase, MAPKKK, and Raf31 in HLB-susceptible accessions; however, these were absent in HLB-tolerant accessions. In contrast, some genes for LRR receptor-like protein kinase (Cs2g10550.1), and serine–threonine protein kinase, plant-type (Cs1g05370.1) were only present in tolerant accessions [7].

A study involving healthy and blight-diseased citrus with *Swingle citrumelo* as the rootstock revealed the involvement of hormone-related genes in response to citrus blight. The up-regulation of salicylic acid and abscisic acid-mediated defense pathways and down-regulation of jasmonic acid- and ethylene-mediated defense pathways were observed in blight-diseased trees [22]. The gene family *NPR1*, which plays a crucial role in mediating the salicylic acid signaling pathway and systemic acquired resistance (SAR) in plants was extensively studied in 10 citrus species. It revealed a total of 63 genes across these species, defining their copy numbers, distribution, phylogenetic groups, evolutionary selection, structural features (introns/exons, motifs, *cis*-regulatory elements), synteny across genomes, and expression patterns in response to bacterial canker [40].

The genome of *P. trifoliata* comprised a family of non-NBS type *LRR* genes that was rapidly evolving. Non-NBS type *LRR* genes and NBS genes located within the disease-resistant QTLs are thought to have provided resistance in *P. trifoliata* against several major pathogens [27]. The NBS-type genes in the *P. polyandra* genome were classified into four groups based on the presence of additional domains, and 17 of those having LRR domains were found to be rapidly evolving [28]. Furthermore, genes for tolerance against HLB, nematodes, CTV (Citrus tristeza virus) have been localized in the *P. polyandra* genome.

### Abiotic stress-related genes

Several gene families were likely to be key attributes of cold resistance in *P. trifoliata*. The gene family of *Tam3-transposase* was found to be rapidly evolving, and the gene *LTI65 (LOW-TEMPERATURE-INDUCED)*, which is a CBF regulon, was found to be positively selected in two mostly cold-tolerant species, *P. trifoliata* and *C. ichangensis* [27]. The *Poncirus polyandra* genome contained 41 gene families associated with the cold signaling pathway, which might contribute to its cold tolerance [28]. The study of Australian wild limes elucidated an expanded number of genes in *C. glauca* related to amino acid and aromatic amino acid biosynthesis and metabolism, suggesting their likely involvement in abiotic stress resistance mechanisms in this species [18, 41–43].

### Physiology and development-related genes

The cytochrome P450 family (CYP), encompassing 260 genes (belonging to 46 clans) involved in metabolism and phytohormone homeostasis, was characterized in satsuma mandarin. The genes in single-family clans were found to be conserved among higher plants, mosses, and algae [24]. The terpenoids are produced via two main biosynthetic pathways: the mevalonic acid (MVA) pathway and the methylerythritol 4-phosphate (MEP) pathway. Studies have uncovered the copy numbers and chromosomal locations of the genes related to these pathways in *C. unshiu* [24], *C. limon* [14], and Australian wild limes [12, 16]. Moreover, these studies also identified genes for pigmentation, such as carotenoids and anthocyanin. The genome of one of the Australian wild limes (*C. australasica*) revealed a significant expansion in thylakoid curvature protein encoding genes (*CURT1*) compared with other Australian citrus and non-Australian citrus, indicating a potential role in conferring tolerance to shade [16]. The copy number and distribution of phloem protein 2 (*PP2*) and callose synthase (*CalS*) gene families were revealed in Australian wild limes, with the highest number of *PP2* and *CalS* genes being identified in *C. glauca* and *C. australasica*, respectively [17].

### Fruiting-related genes

The gene for MADS-RIN, encoding a transcription factor, was found to be a key regulator in fruit ripening in oranges [10]. A key gene encoding d-galacturonic acid reductase (GalUR) regulating the biosynthesis of vitamin C was found to be expanded and the galacturonate pathway was found to be highly up-regulated in orange [10]. The high-quality genome of *C. reticulata* has enabled the detection of positive and negative correlations of many transcription factors with flavonoid-related regulatory and structural gene families, unveiling the regulatory mechanism of flavonoid biosynthesis in *C. reticulata*. Moreover, this study identified the distribution of genes of glycosylated flavonoids (*UGTs*) across the genome, the structural composition of genes, evolutionary relationship of *UGTs*, and expression of *UGTs* in fruit peel at different developmental phases [38]. Key genes for acidity regulation, such as *PH1* and *PH5*, their transcription regulators, and genes involved in citric acid degradation were identified in Australian wild limes [12]. The transcription factor *PH4* was found to induce the expression of *PH1* and *PH5* in citrus, and thereby increase the citric acid levels in citrus. The expression of *PH4* was substantially increased in citrus compared with citrus-related genera. [5].

## Citrus genomes as a model for studying evolutionary events

The genomes have shed light on citrus evolution. The whole-genome duplication events in genomes have been identified using synonymous substitutions (*K*_s_) for paralogous gene pairs in the genome of interest or by orthologous relationships based on inter-genome collinearity. Asian citrus, close citrus relatives, and Australian citrus were found to have no recent whole-genome duplication (WGD) events in addition to the common, ancient triplication (γ-WGT) event [10, 18, 27, 28, 38]. A total of at least 46 or 49 chromosomal fissions and fusion events are thought to have occurred in the evolution of the nine citrus chromosomes to reach the current structure, from the genome structure of the ancient paleohexaploid ancestor [10, 44].

The evolutionary patterns of specific genes or gene families have been largely addressed in plants due to their implications in adaptation and speciation in plants [45]. This has been achieved at whole-genome level with various approaches, such as the likelihood approach estimating the gene family size changes [46], categorizing the unique and shared orthologous gene families [47], and determining the adaptive evolution of gene families with positive or purifying selection through non-synonymous/synonymous (*K*_a_/*K*_s_) ratios of paralogous gene pairs [48]. Gene family analysis revealed 132 significantly expanded gene families in *C. reticulata* cv. ‘Chachi’ (*P* < 0.05), associated with flavonoid biosynthesis, [38], and six significantly expanded gene families in *P. trifoliata* (*P* ≤ 0.01) associated with a group of non-NBS-type LRR proteins, TEs, Tam3-transposase, and a group of zinc finger proteins with the hAT dimerization domain [27] relative to their most recent common ancestors. Furthermore, a high copy number of genes related to an amino acid transporter family was found in cold-tolerant *P. trifoliata* and *F. hindsii*, suggesting that they are likely involved in cold resistance in these two species [27].

Moreover, the expanded gene families in *C. limon* were found to be associated with terpenoid biosynthesis and metabolism, plant–pathogen interactions, stress resistance, and energy metabolism, and may be involved in environmental adaptability [14]. Among the Australian wild limes, *C. glauca* and *C. australasica* had the highest number of expanded gene families (*P* < 0.05). Purine and thiamine metabolism were two significantly expanded gene families in all Australian wild limes. Notably, the expanded gene families in *C. australasica* and *C. australis* were enriched in plant–pathogen interactions [18].

The comparative genome analysis revealed gene conservation (shared gene families) among wild citrus species and cultivated citrus species [26], cultivated citrus [14], citrus and close relatives [27], wild citrus, cultivars and close relatives [33], Australian citrus, and non-Australian citrus [18]. These studies also identified unique gene families in wild citrus (*F. hindsii*) [26], cultivars [14, 33], close relatives (*P. trifoliata*) [27], *P. polyandra* [28], and Australian wild citrus [18] that were enriched with many unique biological processes.

High-quality genomes have facilitated the study of evolutionary relationships among species based on whole-genome-wide single-copy genes. Phylogenetic trees based on single-copy orthologs and *K*_s_-based methods estimated the divergence times among citrus species. As a result, the divergence between *Citrus* and *Poncirus* was estimated to have occurred ~9.8 million years ago (MYA). The divergence between two *Poncirus* species (*P. trifoliata* and *P. polyandra*) was recent and estimated to be within 1.75 and 3.5 MYA [27, 28]. The divergence between *Citrus* and *A. buxifolia* was recently estimated to be 16.39 MYA [33]; however, it had previously been estimated as 5.6–6.4 MYA with corrected evolutionary rates [49]. The estimated divergence times for *C. limon* and *C. medica* (2.85 MYA) [33], *C. clementina* and *C. sinensis* (1.1–1.2 MYA), and *C. clementina* and *C. maxima* (1.8–2.1 MYA) were recent [49]. The Australian citrus and Asian citrus (*C. sinensis*) divergence was estimated to be 8–16 MYA [18] [32].

The selection pressure on all single-copy orthologs of *A. buxifolia*, *C. medica*, *C. ichangensis*, *C. maxima*, *C. clementina*, and *C. sinensis* showed that a majority of genes in *A. buxifolia* had a value of *K*_a_/*K*_s_ > 1, compared with the other species, indicating an increased sequence divergence in *A. buxifolia* relative to the other species [29]. Another study of *P. trifoliata* revealed 35 genes that were positively selected in the *P. trifoliata* lineage [27]. The transcription factor family WRKY is important in mitigating various abiotic and biotic stresses and other physiological processes and has been widely studied in citrus. The WRKY genes in Australian citrus and non-Australian citrus have been found to be primarily evolved through purifying selection in these genomes and expanded through WGD/segmental or tandem duplication events [18, 41–43].

The study by Wu *et al*. [50] revealed important information about the origin, evolution, and domestication of citrus. The study investigated the complex origins of citrus hybrids and admixtures based on SNPs in the genomes. This study identified 10 ancestral citrus species, among which *C. medica*, *C. maxima*, and *C. reticulata* were the progenitor species of several interspecific hybrids, such as lemons, limes, oranges, and grapefruits. The hybrid accessions (including sour orange and lemon) and admixtures (including sweet orange and grapefruits) were identified based on the high segmental heterozygosity and bimodal distributions in heterozygosity in them, respectively. The origin of citrus has been considered to be in Southeast Asia [51], and the progenitor species of citrus are thought to have migrated from Southeast Asia to Australia, giving rise to the Australian limes [50]. A rapid Asian citrus radiation has been found to have occurred in the late Miocene epoch (6–8 MYA) and an Australian citrus radiation has been estimated to have occurred in the early Pliocene epoch (~ 4 MYA). Citrus domestication was found to be associated with an extensive network among mandarins and sweet oranges and a correlation of pummelo admixture with beneficial fruit traits has been found [50].

The domestication of mandarin, which gave rise to existing edible varieties with desirable traits, was known to be primarily associated with apomixis, pummelo introgression, and extensive crosses between ancestral hybrids and admixtures [52]. A decrease in citric acidity was found to be an important trait during mandarin domestication [25]. Two domestication events were known to have happened during mandarin domestication in China, which were characterized by acidity and changes in expression of genes associated with fruit acidity, color, and aroma [25]. Moreover, the origin, evolution, and divergence of pummelo have also been studied using whole-genome sequence data [53].

## Transcriptome analysis

The transcriptome is the complete set of RNA transcript molecules [54]. Transcriptomics initially relied on hybridization-based microarray technologies but these have more recently been replaced by RNA-seq [55]. The availability of high-quality genomes has provided new opportunities for transcriptomics in citrus. Studies have revealed that the genomic introgressions in domesticated citrus (admixed regions) have been associated with regulation of gene expression, resulting in a wide variability of phenotypes in domesticated citrus, such as fruit pulp acidity, easy peeling, and fruit ripening, [56]. This supports the fact that the expression variations of certain genes in admixed regions of the commercial cultivars are linked with domestication. This study further indicated the involvement of differential gene expression in regulating sugar accumulation, organic acid metabolism, pigmentation (carotenoid biosynthesis and degradation), and flavonoid accumulation in citrus. Domestication has also involved promoting the fundamental growth and edible attributes, and a reduction in the production of chemical defenses in citrus plants [57].

Transcriptomic studies have been widely adopted to understand disease resistance/susceptibility in citrus. Studies have shown transcriptomic responses in response to diseases caused by bacteria [58, 59], viruses [60], fungi [61], and peel disorders [62], revealing the pathways involved in various metabolic, cellular, regulatory, and physiological processes in such conditions. The seasonal transcriptomic responses to diseases (impact of day length and temperature on the citrus transcriptome) [63] have also been revealed. Moreover, differentially expressed genes in response to waterlogging conditions [64], water deficit conditions [65], boron deficiency [66], chlorotic conditions [67], different acidity levels [68], citrus dwarfing viroid (CDVd) infection leading to dwarfing phenotype [69], defoliation [70], salinity [71], during fruiting and fruit ripening [72, 73], fruit mastication [74], and self-pruning [75] have been identified.

Furthermore, transcriptomic studies have provided insights into the mechanisms of processes such as low-temperature maintenance of fruit quality during post-harvest storage [76], graft compatibility and incompatibility [77], arbuscular mycorrhizal symbiosis (AMS) [78], citric acid accumulation in pulp and anthocyanin accumulation in the pericarp [79], peel color differences in Australian finger lime [32], gibberellin control of expression of flowering genes [80], the biosynthesis of flavonoids in different fruit tissues at different development stages [23], and polyembryony [29]. Transcriptomes have been used for novel gene discovery [81], to study gene expression at heterozygous loci [82], and in different organs [81] in citrus.

## Epigenetic regulation of citrus genes

The accurate identification of genes in genomes has provided opportunities for studying the epigenetic regulation of certain genes and their impacts on some phenotypes. With regard to citric acid accumulation in fruit pulp, a study has discovered that a reduction in methylation of the *AN1* [encoding a basic helix–loop–helix (bHLH)] promoter leads to an increase in expression, resulting in an accumulation of citric acid in pummelo [79]. Another study revealed the impact of grafting on DNA methylation, where the degree and pattern of DNA methylation were found to depend upon the type of the species and grafting combinations [30]. The grafting combination affects the movement of sRNAs from rootstock to scion, which then mediates the methylation of genes in the scion. Furthermore, the degree of methylation during different phases of lemon development [33], the epigenetic-mediated activation and repression of floral promoters and repressors resulting in alternate bearing [83], and the impact of the degree of methylation in inducing and repressing the transcription of drought-responsive genes [84] have been studied. Methylation effects in response to oomycetes [85] and bacteria [86, 87] have been studied in detail in the last few years.

Moreover, DNA methylation has been found to repress the expression of anthocyanin-related genes in cold storage, resulting in low pigmentation in citrus fruits [88]. Additionally, empirical evidence has been found for DNA hypermethylation-mediated initiation of nucellar polyembryony [89], fruit ripening [90], formation of flowering branches in response to drought [91], and activation of anthocyanin genes in response to fungal infections [92].

## Comparative genomics

### Applications in gene mining and breeding practices

The availability of genome sequences has enabled the mining of desirable genes underlining important physiological and agronomical traits in citrus, which has accelerated genetic breeding in citrus. Apomixis was found to be an important trait in commercial citrus that was selected during domestication [29]. Apomixis was found to be associated with an insertion of a miniature inverted-repeat transposable element (MITE) in the promoter region of the gene *CitRWP*, and the gene was found to have higher expression levels in apomictic cultivars. Polymethoxyflavones (PMFs) are a type of metabolites in citrus with reported anticancer properties, biosynthesized in mandarins and regulated by tandem duplicates of *CreOMT3*, *CreOMT4*, and *CreOMT5* [93]. The modern cultivated mandarins were found to have a 1041-bp deletion in the promoter region of *CreOMT4*, which was associated with a reduced PMF content compared with that in wild and early-admixture mandarins. Two uridine diphosphate (UDP) glycosyltransferases (UGTs) (Ciclev10015105m and Ciclev10019927m) were identified to have a significant correlation with flavonoid biosynthesis, therefore increasing the antioxidants in citrus [94]. The same study revealed the association of PMFs with anticancer effects and the negative impact of coumarins on CYP450 enzymes, increasing the risk of drug interaction. Moreover, the phenotypic diversity between red-flesh and white-flesh pummelos was attributed to the variations in sequence and expression of the gene lycopene cyclase 2 (*LCYB2*). Knowledge of these genes is important in enhancing the beneficial traits and compounds in citrus through breeding approaches.

### Applications in population genetics

Population genetic studies have elucidated the genetic diversity, gene flow, genetic admixture, genetic association with the environment, and beneficial traits of different citrus populations around the world. A study of 18 mandarin populations in Iran has revealed low genetic variability within a single population due to vegetative propagation and apomixis, and high genetic variability among different populations [95]. Other studies have also adopted genetic markers to explore the genetic variability among many citrus species. including mandarins [96, 97], sweet oranges [96], citrons [98], and acid limes [99], and have identified different groups within those populations based on their genetic structures. The analysis of genomes of large populations of citrus species has revealed the hybridization and admixture patterns of citrus cultivars as a part of the domestication process. Accordingly, different admixed mandarin groups, with different degrees of pummelo introgressions [11, 50], sweet oranges and grapefruits as mandarin/pummelo admixtures [100], and limes and lemons as admixtures of two or several ancestral species [101] have been identified. Furthermore, the hybrid origins of grapefruits (pummelo–sweet orange hybrids) and sour oranges (pummelo–mandarin hybrids) have previously been reported [50]. In addition, previous findings have revealed the association of genetic diversity with geography and climatic conditions [95], and phenotypic variations [102]. These studies help enormously in the breeding of citrus with genetic diversity and local adaptations to specific climatic and environmental conditions and will facilitate their conservation.

### A comparison of citrus with other horticultural species revealing the unique features of citrus genomic research

Citrus genomics is unique and distinct compared with other horticultural species due to its unique biology, breeding systems, specific focus on diseases, and economic importance. Citrus species exhibit a complex genetic make-up with a combination of biological and reproductive traits and characteristics, such as a highly heterozygous nature, hybridization, apomixis and nucellar polyembryony, pollen and ovule sterility causing incompatibility, long juvenility, and polyploidy [103, 104]. Although other horticultural species have some of these traits, this combination of characteristics is rare. Citrus species also have a complex domestication history with some cultivars, such as oranges, grapefruits, limes, lemons, and mandarins, showing complex interspecific hybridizations and admixtures of three ancestral species [50]. In contrast, some other horticultural species, including lettuce (*Lactuca sativa*) [105], carrot (*Daucus carota*) [106], pepper (*Capsicum* spp.) [107], and spinach (*Spinacia oleracea*) [108], are known to have a simple domestication history involving a smaller number of ancestral species, minimum genetic changes, and fewer traits selected for human use. Citrus species also show a complex evolutionary history involving many chromosomal changes resulting in a basic chromosome number of 9 that is specific to citrus [44].

Citrus genomic research has devoted considerable efforts to combating diseases that are specific to citrus. The main types of diseases are HLB, citrus canker (bacterial diseases), and CTV virus. A lot of studies have focused on identifying the genetic basis of these diseases, the interactions between vectors and pathogens, the resistance genes in the plants, and other management strategies using genomic approaches [40, 109–111]. Seedlessness is another top priority in citrus due to high consumer preferences and market demands [112], and is less critical in many other horticultural crops, where other traits, such as size and shape of fruits, texture and flavor of fruits, nutritional values, biotic and abiotic stress resistance, high yield, and short juvenility, are among the top-priority breeding goals [113, 114]. Seedlessness in citrus is mainly due to parthenocarpy coupled with self-incompatibility, [115], female and male sterility, and embryo abortion [112], and has been achieved artificially through a variety of genomic approaches, such as ploidy manipulation [116], gamma irradiation [117], hormone application, crossbreeding [118], and gene editing [119]. Citrus genetic research has had a focus on complex biological and reproductive characteristics, specific disease resistance challenges, and other fruit quality traits, such as seedlessness, that have been extensively studied through a combination of conventional and modern genomic approaches. These research strategies have been distinct in citrus compared with other horticultural plants.

## Conclusions, limitations, and future perspectives

Citrus genomes have been improved tremendously since 2013 in terms of their completeness, contiguity, phasing, and reduction in gaps. This has greatly facilitated a significant understanding of gene structures and their precise locations, structural variants, resistant mechanisms towards biotic and abiotic stresses, and the genetic mechanisms underpinning many physiological and developmental processes and evolutionary events. These genomes will also be undoubtedly utilized in future citrus improvement. There is no one powerful assembly method applicable to all genomes due to different complexities, heterozygosities, and repeat contents of different genomes. Therefore, the optimal assembly and annotation pipelines have been tested for these genomes and are continuously being tested and improved. Although phasing was not feasible a few years ago, techniques have been significantly improved to fully resolve haplotypes and to investigate the switching errors between haplotypes. Therefore, monoploid genomes are now being rapidly replaced with haplotype-resolved genomes and have led to many major discoveries about haplotype variations. Among citrus genomes, the largest number of genomes (six) are currently available for *C. sinensis* as it is one of the widely used cultivars worldwide.

Despite the availability of many citrus genomes, this thorough survey identified some gaps and limitations, which might be worth addressing in the future to better understand citrus biology. Although the early genomes for major cultivars have been updated recently with advanced sequencing technologies, there is still only one genome available for *C. clementina*, which was based on Sanger sequencing reads. It can be greatly improved by using the recent sequencing techniques that most of the other genomic studies have used. Although the genomes of Australian wild limes revealed many immunity- and abiotic stress-related genes, they have not yet been associated with phenotypic and expression data pertaining to HLB disease and abiotic stresses, making it impossible to fully understand their resistant mechanisms in response to these conditions. However, the availability of high-quality genomes for Australian limes has provided unprecedented opportunities for future research to study the differential expression and epigenetic-mediated regulatory mechanisms involved in response to such conditions.

Moreover, the development of gapless genomes has been attempted in citrus by filling the gaps in the initial contig assemblies with highly precise HiFi reads and ultra-long ONT reads. The two genomes that have been claimed as gapless were not T2T for all chromosomes, and therefore the question of considering them as gapless arises. The genomes of Australian wild limes were also not T2T (therefore not gapless) due to the presence of large tandem arrays of satellite repeats and extensive ribosomal RNA gene repeats, which could not be spanned by PacBio HiFi reads. The gene completeness of assemblies is primarily determined by sequencing technologies, read coverage, and the assembly strategies used (at contig and scaffolding levels). This survey identified some of the latest genomes with <96% BUSCO scores probably due to low coverage with accurate sequencing and assembly. Some genomes had high coverage of data, however the BUSCO scores were <96%, which might be due to the sequencing accuracy, assembly strategies used and different lineages used for BUSCO analysis. The gene completeness of most of the genomes has been assessed by BUSCO score only, which might not be sufficient, and other measures, such as LTI and CEGMA, could provide more information regarding completeness. The Hi-C data provide long-range linkage information for scaffolding. The recommended Hi-C read depth for genome assemblies is ≥200 million read pairs (400 million reads)/Gb of haploid genome size. The read depths of the sequenced genomes of the Australian wild limes were well beyond that [18]. Despite the high read depths, the Australian genomes depended upon reference assemblies in developing pseudochromosomes. This is due to the fact that Hi-C data alone were not able to assign the contigs into scaffolds for some chromosomes and therefore the generated scaffolds had to be aligned with another reference genome to get more complete assemblies. However, some other species, such as *Mangifera indica* [120], with a similar genome size (~360 Mb), have only used PacBio HiFi reads to get T2T assemblies. The reason for not being able to get T2T assemblies for citrus with PacBio HiFi reads and Hi-C reads with a high coverage of data is primarily due to complex repetitive regions, particularly at the terminal regions of the chromosomes. Therefore, future sequencing programs should focus more on achieving T2T assemblies using, for example, ultra-long ONT reads to close the remaining gaps and to uncover all the important hidden structural elements in these genomes.

Currently haplotype resolution has been performed either by *de novo* or reference-guided methods. The *de novo* assembly-based phasing methods are considered to be more comprehensive, where they use Hi-C signals on initial *de novo* phased contigs for scaffolding. When considering the reference-guided variant phasing methods in citrus, some approaches have used the alignment of contigs against the parental reference genomes to differentiate the haplotypes; however, this method is limited due to the quality of the reference assemblies, repeats, sequencing errors, and depth. In contrast, trio-binning-based phasing (where the long reads of an *F*_1_ hybrid are partitioned into two haplotypes based on parental short reads) is known to perform exceptionally well in achieving highly contiguous and accurately phased contigs [121]. However, the majority of citrus genomes have used Hi-C-based *de novo* phasing due to the lack of parental short reads. Despite their importance, phased genomes have not yet been developed for many important citrus and related genera, including *P. trifoliata*. The phasing of *P. trifoliata* will be important to uncover the haplotype variations underlining the resistance mechanisms against HLB, CTV, *Phytophthora*, and nematodes.

To date, all the available citrus assemblies are diploid. Genomes of the triploid and tetraploid species are not yet available, due to the complexity of assembling higher-ploidy genomes. However, ploidy genomes have been reported for many other plants over the past decade with more recent sequencing methods [122, 123]. High-quality genomes of triploid varieties will be important to better understand the genetic basis of seedlessness, and genomes of other higher polyploids will be important to understand citrus evolution. Moreover, despite the importance of pangenomes, there are only three pangenomes currently available for citrus, which are based on unphased genomes. Pangenomes are important to capture the total genes in a particular species; for example, different accessions of *P. trifoliata* have different degrees of resistance to HLB disease [124]. The development of more phased genomes in the future will enable the construction of more comprehensive pangenomes to better capture the total allelic variants, and allow analysis of allele-specific expression in a particular citrus species or populations.

Based on the available information, citrus genomics research faces several challenges. Citrus species are found to have complex and highly heterozygous genomes, polyploidy, and high levels of complex repetitive regions, which have complicated genomic studies. The integration of epigenomics, transcriptomics, metabolomics, proteomics, and phenomics with genomics to understand trait development is still a significant challenge and is still in its infancy. Traits such as nutritional quality, shelf life, and flavor are regulated by complex genetic mechanisms, which need to be fully understood. Citrus has complex reproductive biological characteristics, such as long juvenility, nucellar polyembryony, different levels of male and female sterility, and self-incompatibility, and other obstacles such as poor seed and pollen development in some cultivars, which have hindered traditional breeding in citrus. Furthermore, conventional hybridization requires 25–30 years to produce commercial cultivars [103]. Moreover, due to the considerable public concerns about the environmental impacts and human health of genetically modified plants having foreign genes from other species, technologies such as gene editing, cisgenesis, and intragenesis need to be used in citrus; however, research on their application is still limited [125].

Future breakthroughs in citrus genomics research could arise from advances in technology, data integration, and new breeding techniques. Long-read sequencing technologies such as PacBio HiFi and ONT, linkage information provided by Hi-C or other technologies may be essential in producing more complete genomes *de novo*, overcoming obstacles related to high heterozygosity and repetitive regions. CRISPR/Cas-based gene editing systems, cisgenesis, intragenesis, and transgrafting technologies will be increasingly adopted in developing varieties with improved biotic and abiotic stress resistance and fruit quality. The validation of candidate genes for desirable traits can be achieved by RNA interference (RNAi) and CRISPR knockouts. Multiomics and single-cell genomics approaches will allow a better understanding of complex traits such as flavor, aroma, diseases, and abiotic stress responses. The prediction of complex traits such as yield, flavor, and disease resistance could be accelerated by artificial intelligence-based tools, and genomic selection would be accelerated by machine learning methods. Moreover, the development of comprehensive databases with well assembled and annotated genomes and other omics data for citrus would be essential for accelerating future citrus research. Improved collaboration among researchers worldwide to share their discoveries and the involvement of growers, breeders, and consumers in collecting accurate data to accelerate trait discovery will be of utmost importance. These strategies will enable the existing challenges in citrus genomics research to be addressed to deliver trait improvement and will ensure more sustainable citrus production to face environmental and market pressures.

## Supplementary Material

Web_Material_uhaf033

## Data Availability

No datasets were generated or analyzed during the current study.

## References

[1] Li C, Lin F, An D. et al. Genome sequencing and assembly by long reads in plants. *Genes*. 2018;9:6 10.3390/genes9010006 PMC5793159 29283420

[2] Kong W, Wang Y, Zhang S. et al. Recent advances in assembly of plant complex genomes. *G7enomics Proteomics*. *Bioinformatics*. 2023;21:427–39 10.1016/j.gpb.2023.04.004 PMC10787022 37100237

[3] Athanasopoulou K, Boti MA, Adamopoulos PG. et al. Third-generation sequencing: the spearhead towards the radical transformation of modern genomics. *Life*. 2022;12:30 10.3390/life12010030 PMC8780579 35054423

[4] Cuenca J, Garcia-Lor A, Navarro L. et al. Citrus genetics and breeding. In: Al-Khayri JM, Jain SM, Johnson DV, eds. Advances in Plant Breeding Strategies: Fruits. Springer: Cham, 2018,403–36

[5] Huang Y, He J, Xu Y. et al. Pangenome analysis provides insight into the evolution of the orange subfamily and a key gene for citric acid accumulation in citrus fruits. *Nat Genet*. 2023;55:1964–75 37783780 10.1038/s41588-023-01516-6

[6] Droc G, Giraud D, Belser C. et al. A super-pangenome for cultivated citrus reveals evolutive features during the allopatric phase of their reticulate evolution. bioRxiv. 2024:2024-10 10.1111/pbi.70553 PMC13110163 41631370

[7] Gao Y, Xu J, Li Z. et al. Citrus genomic resources unravel putative genetic determinants of Huanglongbing pathogenicity. *Iscience*. 2023;26:108255 37927551 10.1016/j.isci.2023.108255 PMC10622705

[8] Zia K, Rao MJ, Sadaqat M. et al. Pangenome-wide analysis of cyclic nucleotide-gated channel (CNGC) gene family in *Citrus* spp. revealed their intraspecies diversity and potential roles in abiotic stress tolerance. *Front Genet*. 2022;13:1034921 36303546 10.3389/fgene.2022.1034921 PMC9593079

[9] Xu Q, Roose ML. Citrus genomes: from sequence variations to epigenetic modifications. In: Gentile A, La Malfa S, Deng Z, eds. The Citrus Genome. Springer: Cham, 2020,141–65

[10] Xu Q, Chen L-L, Ruan X. et al. The draft genome of sweet orange (*Citrus sinensis*). *Nat Genet*. 2013;45:59–66 23179022 10.1038/ng.2472

[11] Wu GA, Prochnik S, Jenkins J. et al. Sequencing of diverse mandarin, pummelo and orange genomes reveals complex history of admixture during citrus domestication. *Nat Biotechnol*. 2014;32:656–62 24908277 10.1038/nbt.2906 PMC4113729

[12] Nakandala U, Masouleh AK, Smith MW. et al. Haplotype resolved chromosome level genome assembly of *Citrus australis* reveals disease resistance and other citrus specific genes. Hortic Res. 2023;10:uhad058 37213680 10.1093/hr/uhad058 PMC10199705

[13] Wang N, Chen P, Xu Y. et al. Phased genomics reveals hidden somatic mutations and provides insight into fruit development in sweet orange. Hortic Res. 2023;11:uhad268 38371640 10.1093/hr/uhad268 PMC10873711

[14] Bao Y, Zeng Z, Yao W. et al. A gap-free and haplotype-resolved lemon genome provides insights into flavor synthesis and huanglongbing (HLB) tolerance. Hortic Res. 2023;10:uhad020 37035858 10.1093/hr/uhad020 PMC10076211

[15] Miao C, Wu Y, Wang L. et al. Haplotype-resolved chromosome-level genome assembly of Huyou (*Citrus changshanensis*). *Sci Data*. 2024;11:605 38849389 10.1038/s41597-024-03437-3 PMC11161639

[16] Nakandala U, Furtado A, Masouleh AK. et al. The genome of *Citrus australasica* reveals disease resistance and other species specific genes. *BMC Plant Biol*. 2024;24:260 38594608 10.1186/s12870-024-04988-8 PMC11005238

[17] Singh K, Huff M, Liu J. et al. Chromosome-scale, de novo, phased genome assemblies of three Australian limes: *Citrus australasica*, *C. inodora*, and *C. glauca*. *Plants*. 2024;**13**:1460. 10.3390/plants13111460 PMC11174732 38891269

[18] Nakandala U, Furtado A, Masouleh AK. et al. The genomes of Australian wild limes. *Plant Mol Biol*. 2024;114:102 39316221 10.1007/s11103-024-01502-4 PMC11422456

[19] Wu B, Yu Q, Deng Z. et al. A chromosome-level phased genome enabling allele-level studies in sweet orange: a case study on citrus Huanglongbing tolerance. Hortic Res. 2023;10:uhac247 36643761 10.1093/hr/uhac247 PMC9832951

[20] Xiong Z, Yin H, Wang N. et al. Chromosome-level genome assembly of navel orange cv. ‘Gannanzao’ (*Citrus sinensis* Osbeck cv. ‘Gannanzao’). *G3 G3 (Bethesda)*. 2024;14:2:uhac247 10.1093/g3journal/jkad268 PMC10849316 38001056

[21] Wang N, Chen P, Xu Y. et al. Phased genomics reveals hidden somatic mutations and provides insight into fruit development in sweet orange. Hortic Res. 2024;11:uhad268 38371640 10.1093/hr/uhad268 PMC10873711

[22] Zhang Y, Barthe G, Grosser JW. et al. Transcriptome analysis of root response to citrus blight based on the newly assembled *Swingle citrumelo* draft genome. *BMC Genomics*. 2016;17:485 27391971 10.1186/s12864-016-2779-y PMC4938905

[23] Zheng W, Zhang W, Liu D. et al. Evolution-guided multiomics provide insights into the strengthening of bioactive flavone biosynthesis in medicinal pummelo. *Plant Biotechnol J*. 2023;21:1577–89 37115171 10.1111/pbi.14058 PMC10363765

[24] Shimizu T, Tanizawa Y, Mochizuki T. et al. Draft sequencing of the heterozygous diploid genome of satsuma (*Citrus unshiu* Marc.) using a hybrid assembly approach. *Front Genet*. 2017;8:180 29259619 10.3389/fgene.2017.00180 PMC5723288

[25] Wang L, He F, Huang Y. et al. Genome of wild mandarin and domestication history of mandarin. *Mol Plant*. 2018;11:1024–37 29885473 10.1016/j.molp.2018.06.001

[26] Zhu C, Zheng X, Huang Y. et al. Genome sequencing and CRISPR/Cas9 gene editing of an early flowering mini-citrus (*Fortunella hindsii*). *Plant Biotechnol J*. 2019;17:2199–210 31004551 10.1111/pbi.13132 PMC6790359

[27] Peng Z, Bredeson JV, Wu GA. et al. A chromosome-scale reference genome of trifoliate orange (*Poncirus trifoliata*) provides insights into disease resistance, cold tolerance and genome evolution in citrus. *Plant J*. 2020;104:1215–32 32985030 10.1111/tpj.14993 PMC7756384

[28] Zhang S, Chen J, Zhang C. et al. Insights into identifying resistance genes for cold and disease stresses through chromosome-level reference genome analyses of *Poncirus polyandra*. *Genomics*. 2023;115:110617 37001742 10.1016/j.ygeno.2023.110617

[29] Wang X, Xu Y, Zhang S. et al. Genomic analyses of primitive, wild and cultivated citrus provide insights into asexual reproduction. *Nat Genet*. 2017;49:765–72 28394353 10.1038/ng.3839

[30] Huang Y, Xu Y, Jiang X. et al. Genome of a citrus rootstock and global DNA demethylation caused by heterografting. *Hortic Res*. 2021;8:69 33790260 10.1038/s41438-021-00505-2 PMC8012640

[31] Guardo MD, Moretto M, Moser M. et al. The haplotype-resolved reference genome of lemon (*Citrus limon* L. Burm f.). Tree Genet Genom. 2021;17:46

[32] Tian Y, Liang T, Peng H. et al. Chromosome-scale genome assembly provides insights into the evolution and color synthesis of finger lemon (*Citrus australasica*). *Tropical Plants*. 2024;3:e015

[33] Yu H, Zhang C, Lu C. et al. The lemon genome and DNA methylome unveil epigenetic regulation of citric acid biosynthesis during fruit development. Hortic Res. 2024;11:uhae005 38464476 10.1093/hr/uhae005 PMC10923643

[34] Ou S, Chen J, Jiang N. Assessing genome assembly quality using the LTR assembly index (LAI). *Nucleic Acids Res*. 2018;46:e126-e. 10.1093/nar/gky730 PMC6265445 30107434

[35] Parra G, Bradnam K, Korf I. CEGMA: a pipeline to accurately annotate core genes in eukaryotic genomes. *Bioinformatics*. 2007;23:1061–7 17332020 10.1093/bioinformatics/btm071

[36] Rhie A, Walenz BP, Koren S. et al. Merqury: reference-free quality, completeness, and phasing assessment for genome assemblies. *Genome Biol*. 2020;21:1–27 10.1186/s13059-020-02134-9 PMC7488777 32928274

[37] Wang L, Huang Y, Liu Z. et al. Somatic variations led to the selection of acidic and acidless orange cultivars. *Nat Plants*. 2021;7:954–65 34140668 10.1038/s41477-021-00941-x

[38] Zhu C, You C, Wu P. et al. The gap-free genome and multi-omics analysis of *Citrus reticulata* ‘Chachi’ reveal the dynamics of fruit flavonoid biosynthesis. *Hortic Res*. 2024;11:uhae177 39108584 10.1093/hr/uhae177 PMC11301317

[39] Gong Y, Li Y, Liu X. et al. A review of the pangenome: how it affects our understanding of genomic variation, selection and breeding in domestic animals? *J Anim Sci*. *Biotechnol*. 2023;14:73 10.1186/s40104-023-00860-1 PMC10161434 37143156

[40] Ali M, Shafiq M, Haider MZ. et al. Genome-wide analysis of NPR1-like genes in citrus species and expression analysis in response to citrus canker (*Xanthomonas axonopodis* pv. *citri*). *Front*. *Plant Sci*. 2024;15:1333286 10.3389/fpls.2024.1333286 PMC11007782 38606070

[41] Ayadi M, Mzid R, Ayed RB. et al. Genome-wide identification and molecular characterization of *Citrus unshiu* WRKY transcription factors in satsuma mandarin: clues for putative involvement in cell growth, fruit ripening, and stress response. *Turk J Agric For*. 2019;43:209–31

[42] Xi D, Yin T, Han P. et al. Genome-wide identification of sweet orange WRKY transcription factors and analysis of their expression in response to infection by *Penicillium digitatum*. *Curr Issues Mol Biol*. 2023;45:1250–71 36826027 10.3390/cimb45020082 PMC9954951

[43] Maheen N, Shafiq M, Sadiq S. et al. Genome identification and characterization of WRKY transcription factor gene family in mandarin (*Citrus reticulata*). *Agriculture*. 2023;13:1182

[44] Feng S, Liu Z, Cheng J. et al. *Zanthoxylum*-specific whole genome duplication and recent activity of transposable elements in the highly repetitive paleotetraploid *Z. bungeanum* genome. Hortic Res. 2021;8:205 34480029 10.1038/s41438-021-00665-1 PMC8417289

[45] Guo YL . Gene family evolution in green plants with emphasis on the origination and evolution of *Arabidopsis thaliana* genes. *Plant J*. 2013;73:941–51 23216999 10.1111/tpj.12089

[46] Mendes FK, Vanderpool D, Fulton B. et al. CAFE 5 models variation in evolutionary rates among gene families. *Bioinformatics*. 2021;36:5516–8 33325502 10.1093/bioinformatics/btaa1022

[47] Sun J, Lu F, Luo Y. et al. OrthoVenn3: an integrated platform for exploring and visualizing orthologous data across genomes. *Nucleic Acids Res*. 2023;51:W397–403 37114999 10.1093/nar/gkad313 PMC10320085

[48] Brookfield J . Evolution: what determines the rate of sequence evolution? *Curr Biol*. 2000;10:R410–1 10837241 10.1016/s0960-9822(00)00506-6

[49] Yuan J, Wang J, Yu J. et al. Alignment of Rutaceae genomes reveals lower genome fractionation level than eudicot genomes affected by extra polyploidization. *Front*. *Plant Sci*. 2019;10:986 10.3389/fpls.2019.00986 PMC6691040 31447866

[50] Wu GA, Terol J, Ibanez V. et al. Genomics of the origin and evolution of *Citrus*. *Nature*. 2018;554:311–6 29414943 10.1038/nature25447

[51] Swingle W, Reece P. History, world distribution, botany, and varieties. In: The Citrus Industry. 2nd ed. University of California press: Berkeley, 1967,190–430

[52] Wu GA, Sugimoto C, Kinjo H. et al. Diversification of mandarin citrus by hybrid speciation and apomixis. *Nat Commun*. 2021;12:4377 34312382 10.1038/s41467-021-24653-0 PMC8313541

[53] Huang Y, Makkumrai W, Fu J. et al. Genomic analysis provides insights into the origin and divergence of fruit flavor and flesh color of pummelo. *New Phytol*. 2024;245:378–91 39526460 10.1111/nph.20223

[54] Yang IS, Kim S. Analysis of whole transcriptome sequencing data: workflow and software. *Genomics Inform*. 2015;13:119–25 26865842 10.5808/GI.2015.13.4.119 PMC4742321

[55] Ozsolak F, Milos PM. RNA sequencing: advances, challenges and opportunities. *Nat Rev Genet*. 2011;12:87–98 21191423 10.1038/nrg2934 PMC3031867

[56] Borredá C, Perez-Roman E, Talon M. et al. Comparative transcriptomics of wild and commercial citrus during early ripening reveals how domestication shaped fruit gene expression. *BMC Plant Biol*. 2022;22:123 35300613 10.1186/s12870-022-03509-9 PMC8928680

[57] Perez-Roman E, Borredá C, Tadeo FR. et al. Transcriptome analysis of the pulp of citrus fruitlets suggests that domestication enhanced growth processes and reduced chemical defenses increasing palatability. *Front*. *Plant Sci*. 2022;13:982683 10.3389/fpls.2022.982683 PMC9478336 36119632

[58] Liu C, Chang X, Li F. et al. Transcriptome analysis of *Citrus sinensis* reveals potential responsive events triggered by *Candidatus* Liberibacter asiaticus. *Protoplasma*. 2024;261:499–512 38092896 10.1007/s00709-023-01911-0

[59] Rodrigues CM, de Souza AA, Takita MA. et al. RNA-Seq analysis of *Citrus reticulata* in the early stages of *Xylella fastidiosa* infection reveals auxin-related genes as a defense response. *BMC Genomics*. 2013;14:676 24090429 10.1186/1471-2164-14-676 PMC3852278

[60] Bin Y, Zhang Q, Su Y. et al. Transcriptome analysis of *Citrus limon* infected with citrus yellow vein clearing virus. *BMC Genomics*. 2023;24:65 36750773 10.1186/s12864-023-09151-5 PMC9903606

[61] Sicilia A, Catara V, Dimaria G. et al. Transcriptome analysis of lemon leaves (Ci*trus limon*) infected by *Plenodomus tracheiphilus* reveals the effectiveness of *Pseudomonas mediterranea* in priming the plant response to mal secco disease. *J Plant Interact*. 2023;18:2243097

[62] Ibáñez AM, Martinelli F, Reagan RL. et al. Transcriptome and metabolome analysis of citrus fruit to elucidate puffing disorder. *Plant Sci*. 2014;217–218:87–98 10.1016/j.plantsci.2013.12.003 24467900

[63] Ribeiro C, Xu J, Hendrich C. et al. Seasonal transcriptome profiling of susceptible and tolerant citrus cultivars to citrus Huanglongbing. *Phytopathology*. 2023;113:286–98 36001783 10.1094/PHYTO-05-22-0179-R

[64] He W, Luo L, Xie R. et al. Transcriptome sequencing analyses uncover mechanisms of citrus rootstock seedlings under waterlogging stress. *Front*. *Plant Sci*. 2023;14:1198930 10.3389/fpls.2023.1198930 PMC10264899 37324702

[65] Romero P, Lafuente MT, Alferez F. Differential transcriptomic regulation in sweet orange fruit (*Citrus sinensis* L. Osbeck) following dehydration and rehydration conditions leading to peel damage. *Front*. *Plant Sci*. 2021;12:732821 10.3389/fpls.2021.732821 PMC8438417 34531889

[66] Yang C-Q, Liu Y-Z, An J-C. et al. Digital gene expression analysis of corky split vein caused by boron deficiency in ‘Newhall’ navel orange (*Citrus sinensis* Osbeck) for selecting differentially expressed genes related to vascular hypertrophy. *PLoS One*. 2013;8:e65737 23755275 10.1371/journal.pone.0065737 PMC3673917

[67] Licciardello C, Torrisi B, Allegra M. et al. A transcriptomic analysis of sensitive and tolerant citrus rootstocks under natural iron deficiency conditions. *J Am Soc Hortic Sci*. 2013;138:487–98

[68] He J, Sun J, Huang Y. et al. Transcriptome analysis reveals the common and specific pathways of citric acid accumulation in different citrus species. *Hortic*. Plant J. 2024; 10.1016/j.hpj.2024.01.003

[69] Lavagi-Craddock I, Dang T, Comstock S. et al. Transcriptome analysis of citrus dwarfing viroid induced dwarfing phenotype of sweet orange on trifoliate orange rootstock. *Microorganisms*. 2022;10:1144 35744662 10.3390/microorganisms10061144 PMC9228058

[70] Dong M, Yin T, Gao J. et al. Transcriptome differential expression analysis of defoliation of two different lemon varieties. *PeerJ*. 2024;12:e17218 38685937 10.7717/peerj.17218 PMC11057431

[71] Asins MJ, Bullones A, Raga V. et al. Combining genetic and transcriptomic approaches to identify transporter-coding genes as likely responsible for a repeatable salt tolerance QTL in citrus. *Int J Mol Sci*. 2023;24:15759 37958745 10.3390/ijms242115759 PMC10650496

[72] Patel M, Manvar T, Apurwa S. et al. Comparative de novo transcriptome analysis and metabolic pathway studies of *Citrus paradisi* flavedo from naive stage to ripened stage. *Mol Biol Rep*. 2014;41:3071–80 24477585 10.1007/s11033-014-3166-x

[73] Shalom L, Samuels S, Zur N. et al. Fruit load induces changes in global gene expression and in abscisic acid (ABA) and indole acetic acid (IAA) homeostasis in citrus buds. *J Exp Bot*. 2014;65:3029–44 24706719 10.1093/jxb/eru148 PMC4071824

[74] Feng G, Ai X, Yi H. et al. Genomic and transcriptomic analyses of *Citrus sinensis* varieties provide insights into Valencia orange fruit mastication trait formation. *Hortic Res*. 2021;8:218 34593784 10.1038/s41438-021-00653-5 PMC8484299

[75] Zhang J-Z, Zhao K, Ai X-Y. et al. Involvements of PCD and changes in gene expression profile during self-pruning of spring shoots in sweet orange (*Citrus sinensis*). *BMC Genomics*. 2014;15:1–16 25308090 10.1186/1471-2164-15-892 PMC4209071

[76] Yun Z, Jin S, Ding Y. et al. Comparative transcriptomics and proteomics analysis of citrus fruit, to improve understanding of the effect of low temperature on maintaining fruit quality during lengthy post-harvest storage. *J Exp Bot*. 2012;63:2873–93 22323274 10.1093/jxb/err390 PMC3350911

[77] He W, Xie R, Wang Y. et al. Comparative transcriptomic analysis on compatible/incompatible grafts in *Citrus*. Hortic Res. 2022;9:uhab072 35043167 10.1093/hr/uhab072 PMC8931943

[78] An J, Sun M, van Velzen R. et al. Comparative transcriptome analysis of *Poncirus trifoliata* identifies a core set of genes involved in arbuscular mycorrhizal symbiosis. *J Exp Bot*. 2018;69:5255–64 30312435 10.1093/jxb/ery283 PMC6184448

[79] Lu Z, Huang Y, Mao S. et al. The high-quality genome of pummelo provides insights into the tissue-specific regulation of citric acid and anthocyanin during domestication. Hortic Res. 2022;9:uhac175 36238347 10.1093/hr/uhac175 PMC9552194

[80] Goldberg-Moeller R, Shalom L, Shlizerman L. et al. Effects of gibberellin treatment during flowering induction period on global gene expression and the transcription of flowering-control genes in *Citrus* buds. *Plant Sci*. 2013;198:46–57 23199686 10.1016/j.plantsci.2012.09.012

[81] Terol J, Tadeo F, Ventimilla D. et al. An RNA-Seq-based reference transcriptome for *Citrus*. *Plant Biotechnol J*. 2016;14:938–50 26261026 10.1111/pbi.12447 PMC11388863

[82] Jiao WB, Huang D, Xing F. et al. Genome-wide characterization and expression analysis of genetic variants in sweet orange. *Plant J*. 2013;75:954–64 23738603 10.1111/tpj.12254

[83] Agustí M, Mesejo C, Muñoz-Fambuena N. et al. Fruit-dependent epigenetic regulation of flowering in *Citrus*. *New Phytol*. 2020;225:376–84 31273802 10.1111/nph.16044

[84] Neves DM, Almeida LAH, Santana-Vieira DDS. et al. Recurrent water deficit causes epigenetic and hormonal changes in citrus plants. *Sci Rep*. 2017;7:13684 29057930 10.1038/s41598-017-14161-x PMC5651809

[85] da Silva AR, da Costa SD, dos Santos Pinto KN. et al. Epigenetic responses to *Phytophthora citrophthora* gummosis in citrus. *Plant Sci*. 2021;313:111082 34763867 10.1016/j.plantsci.2021.111082

[86] Rodrigues M, Nakanishi E, Soutello R. et al. Global methylation in ‘Valencia’ orange seedlings associated with rootstocks and Huanglongbing. *Braz*. *J Biol*. 2023;83:e277679 10.1590/1519-6984.277679 38126644

[87] Fanelli V, De Giovanni C, Saponari M. et al. A possible role of CTV.20 gene methylation in response to *Citrus tristeza virus* infection. *Eur J Plant Pathol*. 2018;150:527–32

[88] Sicilia A, Scialò E, Puglisi I. et al. Anthocyanin biosynthesis and DNA methylation dynamics in sweet orange fruit [*Citrus sinensis* L. (Osbeck)] under cold stress. *J Agric Food Chem*. 2020;68:7024–31 32520546 10.1021/acs.jafc.0c02360 PMC8008385

[89] Jia H-H, Xu Y-T, Yin Z-P. et al. Transcriptomes and DNA methylomes in apomictic cells delineate nucellar embryogenesis initiation in citrus. *DNA Res*. 2021;28:dsab014 34424285 10.1093/dnares/dsab014 PMC8476932

[90] Huang H, Liu R, Niu Q. et al. Global increase in DNA methylation during orange fruit development and ripening. *Proc Natl Acad Sci USA*. 2019;116:1430–6 30635417 10.1073/pnas.1815441116 PMC6347674

[91] Huang B, Wang P, Jin L. et al. Methylome and transcriptome analysis of flowering branches building of *Citrus* plants induced by drought stress. *Gene*. 2023;880:147595 37385391 10.1016/j.gene.2023.147595

[92] Sicilia A, Catara V, Scialò E. et al. Fungal infection induces anthocyanin biosynthesis and changes in DNA methylation configuration of blood orange [*Citrus sinensis* L. (Osbeck)]. *Plants (Basel)*. 2021;**10**:244. 10.3390/plants10020244 PMC7910907 33513740

[93] Peng Z, Song L, Chen M. et al. Neofunctionalization of an OMT cluster dominates polymethoxyflavone biosynthesis associated with the domestication of citrus. *Proc Natl Acad Sci USA*. 2024;121:e2321615121 38530892 10.1073/pnas.2321615121 PMC10998556

[94] Liang X, Wang Y, Shen W. et al. Genomic and metabolomic insights into the selection and differentiation of bioactive compounds in citrus. *Mol Plant*. 2024;17:1753–72 39444162 10.1016/j.molp.2024.10.009

[95] Abbaszadeh M, Sheidai M, Koohdar F. et al. Population and landscape genetic studies in *Citrus tangerina* Tanaka. *Genet Resour Crop Evol*. 2023;70:2695–711

[96] Golein B, Talaie A, Zamani Z. et al. Assessment of genetic variability in some Iranian sweet oranges (*Citrus sinensis* [L.] Osbeck) and mandarins (*Citrus reticulata* Blanco) using SSR markers. *Int J Agric Biol*. 2005;7:167–70

[97] Tapia Campos E, Gutiérrez Espinosa MA, Warburton ML. et al. Characterization of mandarin (*Citrus* spp.) using morphological and AFLP markers. *Interciencia*. 2005;30:687–93

[98] Barbhuiya AR, Khan ML, Dayanandan S. Genetic structure and diversity of natural and domesticated populations of *Citrus medica* L. in the Eastern Himalayan region of Northeast India. *Ecology Evol*. 2016;6:3898–911 10.1002/ece3.2174 PMC4972219 27516853

[99] Shrestha RL, Dhakal DD, Gautum DM. et al. Genetic diversity assessment of acid lime (*Citrus aurantifolia* Swingle*)* landraces in Nepal, using SSR markers. *Am J Plant Sci*. 2012;3:1674–81

[100] Oueslati A, Salhi-Hannachi A, Luro F. et al. Genotyping by sequencing reveals the interspecific *C. maxima*/*C. reticulata* admixture along the genomes of modern citrus varieties of mandarins, tangors, tangelos, orangelos and grapefruits. *PLoS One*. 2017;12:e0185618 28982157 10.1371/journal.pone.0185618 PMC5628881

[101] Curk F, Ollitrault F, Garcia-Lor A. et al. Phylogenetic origin of limes and lemons revealed by cytoplasmic and nuclear markers. *Ann Bot*. 2016;117:565–83 26944784 10.1093/aob/mcw005 PMC4817432

[102] Neves CG, Do Amaral DOJ, de Paula MFB. et al. Characterization of tropical mandarin collection: implications for breeding related to fruit quality. *Sci Hortic*. 2018;239:289–99

[103] Caruso M, Smith MW, Froelicher Y. et al. Traditional breeding. In: Talon M, Caruso M, Gmitter FG, eds. The Genus Citrus. Woodhead Publishing, Sawston; 2020,129–48

[104] Aleza P, Garavello MF, Rouiss H. et al. Inheritance pattern of tetraploids pummelo, mandarin, and their interspecific hybrid sour orange is highly influenced by their phylogenomic structure. *Front*. *Plant Sci*. 2023;14:1327872 10.3389/fpls.2023.1327872 PMC10739408 38143579

[105] Wei T, Van Treuren R, Liu X. et al. Whole-genome resequencing of 445 *Lactuca* accessions reveals the domestication history of cultivated lettuce. *Nat Genet*. 2021;53:752–60 33846635 10.1038/s41588-021-00831-0

[106] Ellison S . Carrot domestication. In: Simon P, Iorizzo M, Grzbelus D. et al., eds. The Carrot Genome. Springer, New York; 2019,77–91

[107] Liu F, Zhao J, Sun H. et al. Genomes of cultivated and wild *Capsicum* species provide insights into pepper domestication and population differentiation. *Nat Commun*. 2023;14:5487 37679363 10.1038/s41467-023-41251-4 PMC10484947

[108] Ribera A, van Treuren R, Kik C. et al. On the origin and dispersal of cultivated spinach (*Spinacia oleracea* L.). *Genet Resour Crop Evol*. 2021;68:1023–32

[109] Asins MJ, Bernet GP, Ruiz C. et al. QTL analysis of citrus tristeza virus-citradia interaction. *Theor Appl Genet*. 2004;108:603–11 14614564 10.1007/s00122-003-1486-7

[110] Huang C-Y, Araujo K, Sánchez JN. et al. A stable antimicrobial peptide with dual functions of treating and preventing citrus Huanglongbing. *Proc Natl Acad Sci USA*. 2021;118:e2019628118 33526689 10.1073/pnas.2019628118 PMC8017978

[111] Killiny N . Made for each other: vector–pathogen interfaces in the huanglongbing pathosystem. *Phytopathology*. 2022;112:26–43 34096774 10.1094/PHYTO-05-21-0182-FI

[112] Yin P, Ding W, Zhang H. et al. Morphological, physiological and molecular characteristics of the seedless ‘Hongjiangcheng’ sweet orange. *Hortic Plant J*. 2023;9:437–49

[113] Bura S, Jasrotia A, Sharma S. et al. Recent advances in breeding of mango (*Mangifera indica*): a review. *Int J Environ Climate Change*. 2023;13:521–38

[114] Kole C . Fruits and Nuts. Springer Science & Business Media; New York; 2007:

[115] Vardi A, Levin I, Carmi N. Induction of seedlessness in citrus: from classical techniques to emerging biotechnological approaches. *J Am Soc Hortic Sci*. 2008;133:117–26

[116] Ollitrault P, Dambier D, Luro F. et al. Ploidy manipulation for breeding seedless triploid citrus. In: Janick J, ed. ed.Plant Breeding Reviews, Vol. 30. Wiley, 2007,323–52

[117] Goldenberg L, Yaniv Y, Porat R. et al. Effects of gamma-irradiation mutagenesis for induction of seedlessness, on the quality of mandarin fruit. *Food Nutr Sci*. 2014;05:943–52

[118] Moniruzzaman M, Darwish AG, Ismail A. et al. Seedlessness trait and genome editing—a review. *Int J Mol Sci*. 2023;24:5660 36982733 10.3390/ijms24065660 PMC10057249

[119] Poles L, Ciacciulli A, Pappalardo H. et al. Genome editing of IKU1 to obtain citrus seedless fruits. Acta Hortic. 2024;1399:139–44

[120] Wijesundara UK, Masouleh AK, Furtado A. et al. A chromosome-level genome of mango exclusively from long-read sequence data. *Plant*. *Genome*. 2024;17:e20441 10.1002/tpg2.20441 PMC12806855 38462715

[121] Koren S, Rhie A, Walenz BP. et al. De novo assembly of haplotype-resolved genomes with trio binning. *Nat Biotechnol*. 2018;36:1174–82 10.1038/nbt.4277 PMC6476705 30346939

[122] Hoopes G, Meng X, Hamilton JP. et al. Phased, chromosome-scale genome assemblies of tetraploid potato reveal a complex genome, transcriptome, and predicted proteome landscape underpinning genetic diversity. *Mol Plant*. 2022;15:520–36 35026436 10.1016/j.molp.2022.01.003

[123] Healey A, Garsmeur O, Lovell J. et al. The complex polyploid genome architecture of sugarcane. *Nature*. 2024;628:804–10 38538783 10.1038/s41586-024-07231-4 PMC11041754

[124] Alves MN, Lopes SA, Raiol-Junior LL. et al. Resistance to ‘*Candidatus* Liberibacter asiaticus,’ the huanglongbing associated bacterium, in sexually and/or graft-compatible *Citrus* relatives. *Front* *Plant Sci*. 2021;11:2166. 10.3389/fpls.2020.617664 PMC7820388 33488659

[125] Conti G, Xoconostle-Cázares B, Marcelino-Pérez G. et al. Citrus genetic transformation: an overview of the current strategies and insights on the new emerging technologies. *Front*. *Plant Sci*. 2021;12:2519 10.3389/fpls.2021.768197 PMC8670418 34917104

