# Supplementary material for: Citrus genomes: past, present and future
Source: Hortic Res. 2025 Feb 4;12(5):uhaf033. doi: 10.1093/hr/uhaf033 (PMC11992330; doi:10.1093/hr/uhaf033)
Supplement: Web_Material_uhaf033 [file web_material_uhaf033.zip › Supplementary Table S1_Clean version.docx]

**Supplementary Table S1 Sequencing and assembly approaches and outcomes for available genomes of domesticated and wildcitrus wild species and citrus relatives**

| **Common name** | **Scientific name** | **Ploidy** | **Domesticated status** | **Sequencing platforms and mapping techniques** | **Genome assembly tools** | **Estimated genome size (Mb)** | **Assembly contiguity (N50) (Mb) and completeness (BUSCO) and CEGMA (%)** | **Chromosome level (CL) / Draft assembly (D), Haplotypes resolved (HR) / Haplotypes not resolved (HNR), Telomeres reported (TR) / Telomeres not reported (TNR)** | **Reference** |
| --- | --- | --- | --- | --- | --- | --- | --- | --- | --- |
| Valencia Sweet orange | *C. Sinensis* cv. Valencia | Di-haploid | Cultivar | Illumina, genetic maps | SOAPdenovo & Opera | 367.0 | Scaffold N50: 1.69 | CL, HNR, TNR | (1) |
| Sweet orange | *C. Sinensis* | Di-haploid | Cultivar | PacBio RSII, ONT, Illumina | HGAP, quiver and pilon, SSPACE, GapCloser package | - | Contig N50: 24.2  Assembly BUSCO: > 90 | CL, HNR, TNR | (2) |
| Valencia sweet orange (DVS) | *C. Sinensis cv.* Valencia | Diploid | Cultivar | PacBio CLR | MECAT2, CANU, Falcon | - | Contig N50: 15.4  Assembly BUSCO: 98.7,  QV: 50.6 | CL, HR, TNR | (3) |
| Navel orange | *C. sinensis Osbeck cv. Gannanzao* | Diploid | Cultivar | PacBio HiFi, Hi-C | Hifiasm, LACHESIS | 334.7 | Scaffold N50: 31.86  Assembly BUSCO: 94.6  CEGMA score: 93.55 | CL, HNR, TNR | (4) |
| Jinhong sweet orange | *C. Sinensis* cv. Jinhong | Diploid | Cultivar | PacBio HiFi, ONT, Hi-C | Hifiasm, Ragtag, 3D-DNA | - | Contig N50: 20.6, 16.4  Assembly BUSCO: > 98.5 | CL, HR, TNR | (5) |
| Newhall navel orange | *C. sinensis* cv. *‘Newhall’* | Diploid | cultivar | PacBio HiFi, Hi-C, Illumina | HiFiasm, ALLHIC, Juicebox | 350.0 | Contig N50: 12.5  Scaffold N50: 32.8  Assembly BUSCO: 95.1 | CL, HR, TNR | (6) |
| Clementine mandarin | *C. clementina* | Haploid | Cultivar | Sanger, genetic maps | Arachne | 301.4 | - | CL, HNR, TNR | (7) |
| Citrumelo | *Swingle citrumelo* |  |  | Illumina | CLC genomic workbench  v6.0.1 | 380.0 | Scaffold N50: 11.4 kb  CEGMA score: 96.3 | D, HNR, TNR | (8) |
| Pummelo | *C. maxima / C. grandis* | Diploid | Cultivar | PacBio RSII & Illumina | SOAPdenovo package | 345.8 | Scaffold N50: 4.2 | CL, HNR, TNR | (9) |
| Pummelo | *C. maxima / C. grandis* | Diploid | Cultivar | ONT, Hi-C | NECAT, Racon, Nextpolish, Juicer, 3D-DNA | 367.6 | Contig N50: 3.8  Scaffold N50: 38.7  Assembly BUSCO: 98.1 | CL, HNR, TNR | (10) |
| Pummelo | *C. maxima / C. grandis* | Diploid | Cultivar | Illumina, PacBio CLR, Hi-C | Canu, Minimap2, purge_dups, Nextpolish, ALLHiC, MUMMER4 | 349.0 | Contig N50: 1.74  Assembly BUSCO: 99.1 | CL, HNR, TNR | (11) |
| Citron | *C. medica* | Diploid | Wild | Illumina | SOAPdenovo package | - | - | D, HNR, TNR | (9) |
| Papeda | *C. ichangensis* | Diploid | Wild | Illumina | SOAPdenovo package | - | - | D, HNR, TNR | (9) |
| Atalantia/Chinese box orange | *Atalantia buxifolia* | Diploid | Wild | Illumina | SOAPdenovo package | - | - | D, HNR, TNR | (9) |
| Satsuma mandarin | *C. unshiu* Marc. | Diploid | Cultivar | Illumina & PacBio RSII | PLATANUS, Opera & PBJelly | 359.7 | Scaffold N50: 0.39  Assembly BUSCO: 94.2  Annotation BUSCO: 92.1 | CL, HNR, TNR | (12) |
| Mangshan wild mandarin | *C. reticulata* | Diploid | Wild | Illumina | PLATANUS & GapCloser software | - | Scaffold N50: 1.7  Assembly BUSCO: 96 | CL, HNR, TNR | (13) |
| Dazhongyoushen | *C. reticulata* cv. Chachi | Diploid | Cultivar | ONT, MGI short reads | NextDenovo, NextPolish, purge_dups, | 313.0 | Contig N50: 30.2  Scaffold N50: 32.2  Assembly BUSCO: 98.4 | CL, HNR, TR | (14) |
| Hongkong kumquat | *Fortunella hindsii* | Diploid | Wild | PacBio CLR & Illumina, 10X genomic data | Mecat, ARCS and LINKS | 374.0 | Scaffold N50: 5.2  Assembly BUSCO: 95.1 | D, HNR, TNR | (15) |
| Hongkong kumquat | *F. hindsii* | Diploid | Wild | PacBio CLR, Illumina, Hi-C | Canu, Smartdenovo, LACHESIS | 323.72 | Contig N50 – 9.77  BUSCO – 97% | CL, HNR, TNR | (16) |
| Trifoliate orange | *Poncirus trifoliata* | Diploid | Close relative | PacBio CLR, Illumina, Hi-C | Falcon, HiRise, Juicebox | 264.9 | Scaffold N50: 27.7  Assembly BUSCO: 97.2 | CL, HNR, TNR | (17) |
| Trifoliate orange | *P. trifoliata* | Diploid | Close relative | PacBio RSII, Illumina | Falcon/Falcon_  Unzip, Quiver, Pilon | 335.0 | Contig N50: 1.17  Assembly BUSCO: 97.4 | CL, HNR, TNR | (18) |
| - | *P. polyandra* | Diploid | Close relative | ONT, Hi-C | NextDenovo, Racon, NextPolish, LACHESIS | 315.8 | Contig N50: 7.57  Scaffold N50: 32.07  Assembly BUSCO: 98.82 | CL, HNR, TNR | (19) |
| Lemon | *C. limon* | Diploid | Cultivar | ONT, Illumina | MaSuRCA v.3.4.1; falcon build 180808 | 312.8  324.7 | Hap1 scaffold N50: 27.1  Hap2 scaffold N50: 28.4  Hap1 assembly BUSCO: 95.9  Hap2 assembly BUSCO: 94.8 | CL, HR, TNR | (20) |
|  |  |  |  |  |  |  |  |  |  |
| Lemon | *C. limon* | Diploid | Cultivar | PacBio CCS, ONT, Hi-C | Hifiasm, Verkko, ALLHiC, TGS-GapCloser | - | Contig N50: 35.6  Assembly BUSCO: 98.7  QV of individual chr: 62 - 81 | CL, HR, TR | (21) |
| lemon | *C. limon* | Diploid | Cultivar | PacBio, Illumina, Hi-C | Canu, Pilon, LAchesis | 380.1 | Contig N50 – 3  Scaffold N50 – 38.6  Assembly BUSCO – 95  CEGMA score: 97.18 | CL, HNR, TNR | (22) |
| Australian round lime | *C. australis* | Diploid | Wild | PacBio CCS, Hi-C | HiFiasm, SALSA | 328.5  (Collapsed), 325.8 (hap1), 300 (hap2) | Scaffold N50 – 30.7–35.1  BUSCO – 97.4-98.8 | CL, HR, TR | (23) |
| Citrus pangenome | *C. mangensis, C. linwuensis, C. ichangensis, C. australasica, C. hongheensis, C. maxima 'Majia',*  *Murraya paniculata, Clausena lansium, Luvunga scandens, Aegle marmelos, Citropsis gilletiana, Atalantia buxifolia* | Diploid | Citrus and citrus-related species | PacBio, ONT, Hi-C | SMRTdenovo, miniasm, nextdenovo, necat, canu, Racon, nextpolish, 3D-DNA | 217.8 to 419.1 | Contig N50 – 1.6-16.8 | A. buxifolia and *C. linwuensis*: CL, Others: D, All genomes: HNR, TNR | (24) |
| Australian finger lime | *C. australasica* cv. Rainbow | Diploid | Wild | PacBio CCS (HiFi), Hi-C | HiFiasm, BWA + Arima mapping + SALSA | 344.2  (Collapsed), 321.1 (hap1), 323.2 (hap2) | Scaffold N50 – 32.3–35  BUSCO – 98.9-99.1 | CL, HR, TR | (25) |
| Australian finger lime | *C. australasica* | Diploid | Wild | PacBio CCS, Hi-C | HiFiasm, FALCON-Phase, Phase Genomics’ Proximo Hi-C genome scaffolding platform | 337 .0 (primary)  335 .0 (alternate) | Scaffold N50 – 30 - 30.2  BUSCO - 98.70–99.20% | CL, HR, TNR | (26) |
| Australian finger lime | *C. australasica* cv. YaoJi | Haploid | Wild | ONT | Nextdenovo, Nextpolish, Ragtag | 314.6 | Contig N50: 6.6  Scaffold N50: 32.8  QV: 27.68  LAI: 15.19 | CL, HNR, TNR | (27) |
| Russell River lime | *C. inodora* | Diploid | Wild | PacBio CCS, Hi-C | HiFiasm, BWA + Arima mapping + SALSA | 391.1  (Collapsed), 290.8 (hap1), 325.3 (hap2) | Scaffold N50 – 29.3–31.5  BUSCO – 98.8-99.0 | CL, HR, TR | (28) |
| Russell River lime | *C. inodora* | Diploid | Wild | PacBio CCS, Hi-C | HiFiasm, FALCON-Phase, Phase Genomics’ Proximo Hi-C genome scaffolding platform | 303.7 (primary)  298.8 (alternate) | Scaffold N50 – 28.9  BUSCO - 96.68–96.70% | CL, HR, TNR | (26) |
| Dessert lime | *C. glauca* | Diploid | Wild | PacBio CCS, Hi-C | HiFiasm, BWA + Arima mapping + SALSA | 340.2  (Collapsed), 318 (hap1), 311.6 (hap2) | Scaffold N50 – 32.4–33.5  BUSCO – 98.4-99.5 | CL, HR, TR | (28) |
| Dessert lime | *C. glauca* | Diploid | Wild | PacBio CCS, Hi-C | HiFiasm, FALCON-Phase, Phase Genomics’ Proximo Hi-C genome scaffolding platform | 376.4 (primary)  379.2 (alternate) | Scaffold N50 – 36.7, 36.9  BUSCO - 96.90–97.20% | CL, HR, TNR | (26) |
| Mount white lime | *C. garrawayi* | Diploid | Wild | PacBio CCS, Hi-C | HiFiasm, BWA + Arima mapping + SALSA | 316.8  (Collapsed), 286.8 (hap1), 312.7 (hap2) | Scaffold N50 – 28.9–30  BUSCO – 98.3-98.6 | CL, HR, TR | (28) |
| Huyou | *C. changshanensis* | Diploid | Landrace | Illumina, PacBio HiFi, Hi-C | HiFiasm, 3D-DNA | 354.6 | Contig N50 – 30.1 - 32.4  Assembly BUSCO – 98-98.2  QV: 42.74 – 43.26  LAI: 15.4 – 19.54 | CL, HR, TNR | (29) |
| Citrus pangenome | *C. reticulata, C. medica and C. micrantha* | Diploid | Cultivar | Illumina, ONT, Bionano optical mapping | Necat, HAPO-G, BiSCoT | 332.1 – 346.4 | Assembly BUSCO > 98.4 | CL, HNR, TNR | (30) |

*PacBio (Pacific Bioscience), ONT (Oxford Nanopore Technology), CCS - Circular Consensus Sequencing, CLR - Continuous Long Reads, BiSCoT - Bionano Scaffolding Correction Tool, QV – Quality value, LAI – LTR assembly index, CEGMA (Core Eukaryotic Genes Mapping Approach).

**References**

1. Xu Q, Chen L-L, Ruan X, Chen D, Zhu A, Chen C, et al. The draft genome of sweet orange (Citrus sinensis). Nat Genet. 2013;45(1):59-66.

2. Wang L, Huang Y, Liu Z, He J, Jiang X, He F, et al. Somatic variations led to the selection of acidic and acidless orange cultivars. Nature Plants. 2021;7(7):954-65.

3. Wu B, Yu Q, Deng Z, Duan Y, Luo F, Gmitter Jr F. A chromosome-level phased genome enabling allele-level studies in sweet orange: a case study on citrus Huanglongbing tolerance. Horticulture Research. 2023;10(1):uhac247.

4. Xiong Z, Yin H, Wang N, Han G, Gao Y. Chromosome-level genome assembly of navel orange cv. Gannanzao (Citrus sinensis Osbeck cv. Gannanzao). G3 Genes|Genomes|Genetics. 2023.

5. Wang N, Chen P, Xu Y, Guo L, Li X, Yi H, et al. Phased genomics reveals hidden somatic mutations and provides insight into fruit development in sweet orange. Horticulture Research. 2024;11(2):uhad268.

6. Gao Y, Xu J, Li Z, Zhang Y, Riera N, Xiong Z, et al. Citrus genomic resources unravel putative genetic determinants of Huanglongbing pathogenicity. Iscience. 2023;26(2).

7. Wu GA, Prochnik S, Jenkins J, Salse J, Hellsten U, Murat F, et al. Sequencing of diverse mandarin, pummelo and orange genomes reveals complex history of admixture during citrus domestication. Nat Biotechnol. 2014;32(7):656-62.

8. Zhang Y, Barthe G, Grosser JW, Wang N. Transcriptome analysis of root response to citrus blight based on the newly assembled Swingle citrumelo draft genome. BMC Genomics. 2016;17(1):1-10.

9. Wang X, Xu Y, Zhang S, Cao L, Huang Y, Cheng J, et al. Genomic analyses of primitive, wild and cultivated citrus provide insights into asexual reproduction. Nat Genet. 2017;49(5):765-72.

10. Lu Z, Huang Y, Mao S, Wu F, Liu Y, Mao X, et al. The high-quality genome of pummelo provides insights into the tissue-specific regulation of citric acid and anthocyanin during domestication. Horticulture Research. 2022;9:uhac175.

11. Zheng W, Zhang W, Liu D, Yin M, Wang X, Wang S, et al. Evolution‐guided multiomics provide insights into the strengthening of bioactive flavone biosynthesis in medicinal pummelo. Plant Biotechnol J. 2023.

12. Shimizu T, Tanizawa Y, Mochizuki T, Nagasaki H, Yoshioka T, Toyoda A, et al. Draft sequencing of the heterozygous diploid genome of Satsuma (Citrus unshiu Marc.) using a hybrid assembly approach. Frontiers in genetics. 2017;8:180.

13. Wang L, He F, Huang Y, He J, Yang S, Zeng J, et al. Genome of wild mandarin and domestication history of mandarin. Molecular plant. 2018;11(8):1024-37.

14. Zhu C, You C, Wu P, Huang Y, Zhang R, Fan Z, et al. The gap-free genome and multi-omics analysis of Citrus reticulata ‘Chachi’reveal the dynamics of fruit flavonoid biosynthesis. Horticulture Research. 2024;11(8):uhae177.

15. Zhu C, Zheng X, Huang Y, Ye J, Chen P, Zhang C, et al. Genome sequencing and CRISPR/Cas9 gene editing of an early flowering Mini‐Citrus (Fortunella hindsii). Plant Biotechnol J. 2019;17(11):2199-210.

16. Wang N, Song X, Ye J, Zhang S, Cao Z, Zhu C, et al. Structural variation and parallel evolution of apomixis in citrus during domestication and diversification. National Science Review. 2022;9(10):p.nwac114.

17. Peng Z, Bredeson JV, Wu GA, Shu S, Rawat N, Du D, et al. A chromosome‐scale reference genome of trifoliate orange (Poncirus trifoliata) provides insights into disease resistance, cold tolerance and genome evolution in Citrus. The Plant Journal. 2020;104(5):1215-32.

18. Huang Y, Xu Y, Jiang X, Yu H, Jia H, Tan C, et al. Genome of a citrus rootstock and global DNA demethylation caused by heterografting. Horticulture research. 2021;8(1):1-13.

19. Zhang S, Chen J, Zhang C, Zhang S, Zhang X, Gao L, et al. Insights into identifying resistance genes for cold and disease stresses through chromosome-level reference genome analyses of Poncirus polyandra. Genomics. 2023;115(3):110617.

20. Guardo MD, Moretto M, Moser M, Catalano C, Troggio M, Deng Z, et al. The haplotype-resolved reference genome of lemon (Citrus limon L. Burm f.). Tree Genet Genom. 2021;17(6):1-12.

21. Bao Y, Zeng Z, Yao W, Chen X, Jiang M, Sehrish A, et al. A gap-free and haplotype-resolved lemon genome provides insights into flavor synthesis and huanglongbing (HLB) tolerance. Horticulture Research. 2023;10(4):uhad020.

22. Yu H, Zhang C, Lu C, Wang Y, Ge C, Huang G, et al. The lemon genome and DNA methylome unveil epigenetic regulation of citric acid biosynthesis during fruit development. Horticulture Research. 2024;11(3):uhae005.

23. Nakandala U, Masouleh AK, Smith MW, Furtado A, Mason P, Constantin L, et al. Haplotype resolved chromosome level genome assembly of Citrus australis reveals disease resistance and other citrus specific genes. Horticulture Research. 2023;10(5):uhad058.

24. Huang Y, He J, Xu Y, Zheng W, Wang S, Chen P, et al. Pangenome analysis provides insight into the evolution of the orange subfamily and a key gene for citric acid accumulation in citrus fruits. Nat Genet. 2023:1-12.

25. Nakandala U, Furtado A, Masouleh AK, Smith MW, Williams DC, Henry RJ. The genome of Citrus australasica reveals disease resistance and other species specific genes. BMC Plant Biol. 2024;24(1):260.

26. Singh K, Huff M, Liu J, Park J-W, Rickman T, Keremane M, et al. Chromosome-Scale, De Novo, Phased Genome Assemblies of Three Australian Limes: Citrus australasica, C. inodora, and C. glauca. Plants. 2024;13(11):1460.

27. Tian Y, Liang T, Peng H, Wang Q, Luo X, Xu R, et al. Chromosome-scale genome assembly provides insights into the evolution and color synthesis of finger lemon (Citrus australasica). Tropical Plants. 2024;3(1).

28. Nakandala U, Furtado A, Masouleh AK, Smith MW, Mason P, Williams DC, et al. The genomes of Australian wild limes. Plant Mol Biol. 2024;114(5):102.

29. Miao C, Wu Y, Wang L, Zhao S, Grierson D, Xu C, et al. Haplotype-resolved chromosome-level genome assembly of Huyou (Citrus changshanensis). Scientific Data. 2024;11(1):605.

30. Droc G, Giraud D, Belser C, Labadie K, Duprat S, Cruaud C, et al. A super-pangenome for cultivated citrus reveals evolutive features during the allopatric phase of their reticulate evolution. bioRxiv. 2024:2024.10. 17.618847.
