# Supplementary material for: Citrus genomes: past, present and future
Source: Hortic Res. 2025 Feb 4;12(5):uhaf033. doi: 10.1093/hr/uhaf033 (PMC11992330; doi:10.1093/hr/uhaf033)
Supplement: Web_Material_uhaf033 [file web_material_uhaf033.zip › Supplemnetary Table S2_Clean version.docx]

| **Supplementary Table S2** Annotation (structural and functional) of citrus genomes | | | | | |  | | | | |  |
| --- | --- | --- | --- | --- | --- | --- | --- | --- | --- | --- | --- |
| **Genome** | **Repeat analysis** | |  | **Gene prediction** | | | | | **Functional annotation** | **Reference** | |
|  | **Repeat detection and masking tools** | **Total repeat composition (%)** |  | **Experimental and other external evidence** | **Evidence (RNA-seq) alignment tools** | | **Gene prediction tools** | **Total genes in the assembled genome** |  |  |  |
| *C. sinensis* | LTR elements: LTRharvest, findltr, DNA transposons and LINEs: TransposonPSI, Novel TE elements: RepeatScout, Repeatmasker | 20.5 |  | EST, RNA-Seq, RNA-PET, Proteins | PASA | | Ab initio tools (Fgenesh, GeneID, Genscan and GlimmerHMM) | 29,445  (23,421 in pseudochromosomes) | BLASTP (E value < 10−5) against Uniprot database (including the SWISS-PROT and TrEMBL databases) | (1) | |
| *C. sinensis* | RepeatModeler, Repeatmasker | - |  | EST, RNA-seq, Proteins | - | | Ab initio tools (AUGUSTUS & GlimmerHMM),  Homology searches (exonerate & AAT), transcriptome assembly (Trinity), combined all gene structures (EVM) | 29,875 | - | (2) | |
| *C. sinensis* | RepeatModeler, EDTA, Repeatmasker | - |  | RNA-seq, Proteins | HISAT2 | | BRAKER v2.1.5 | Total genes – 55,745 (99.2%), DVS_A – 27,807,  DVS_B – 27,938 | - | (3) | |
| *C. sinensis* | TRF, Repbase, LTR_FINDER, RepeatScout, RepeatModeler, Repeatmasker | 49.29 |  | RNA-seq, Proteins | HISAT2 | | Ab initio tools (Augustus, Geneid, Genescan, GlimmerHMM, SNAP), Homology-based prediction (GeneWise), transcriptome assembly (Trinity), combined all gene structures (EVM) | 23,037 (96.8%) | BLASTP (E-value ≤ 1e−5) against Swiss-Prot and NR databases, motifs and domains (InterProScan), Gene Ontology ID assignment, Pathway analysis (KEGG) | (4) | |
| *C. sinensis* | EDTA, TRF | Total TE composition (46.17 & 44.77 for two haplotypes) |  | RNA-seq, proteins | STAR | | Ab initio tools (Augustus, SNAP), Maker | 30,908 (Haplotype A), 29,913 (Haplotype B) | - | (5) | |
| *C. sinensis* | TRF, LTR_FINDER, RepeatScout, RepeatModeler, Repbase, Repeatmasker | 52 |  | RNA-seq, proteins | HISAT2 | | Ab initio tools (Augustus, Geneid, Genescan, GlimmerHMM, SNAP), Homology searches (GeneWise), transcriptome assembly (Trinity), combined all gene structures (EVM) | 46,616 (One haplotype - 22,916, other haplotype - 22,824) | BLASTP (E-value ≤ 1e−5) against Swiss-Prot and NR databases, motifs and domains (InterProScan), Gene Ontology ID assignment, Pathway analysis (KEGG) | (6) | |
| *C. clementina* | RepeatModeler, Repeatmasker | 45 |  | EST | PASA | | Ab initio tools (Fgenesh, exonerate & GenomeScan) | ~25,000 | - | (7) | |
| *Swingle citrumelo* | RepeatModeler, Repeatmasker, MAKER2 internal RepeatRunner package | 44.8 |  | EST, alt-EST & RNA-seq | TOPHAT2 | | MAKER2 (SNAP, AUGUSTUS and GeneMark) | 29,054 | BLASTP (E-value ≤ 1e−5) against Swiss-Prot | (8) | |
| *C. grandis* | RepeatModeler, TIGR, TREP, C.  sinensis repeat sequence database, TEClass software, Repeatmasker | - |  | EST, RNA-seq, Proteins | - | | Ab initio tools (AUGUSTUS, GlimmerHMM & SNAP), homology search (exonerate, AAT) | 30,123 | BLASTP (SwissProt and TrEMBL), motifs and domains (InterProScan) | (9) | |
| *C. grandis* | RepeatModeler, Repbase, Repeatmasker | 57.07 |  | RNA-seq, Proteins | HISAT2, PASA | | Ab initio tools (Augustus, GlimmerHMM), homology search (GenomeThreader), combined all gene structures (EVM) | 26,988 | - | (10) | |
| *C. grandis* | RepeatModeler, Repeatmasker | - |  | RNA-seq, Proteins | PASA | | Ab initio tools (AUGUSTUS, GlimmerHMM, SNAP), homology search (GenomeThreader), combined all gene structures (EVM) | 26,924 | - | (11) | |
| *C. medica* | RepeatModeler, TIGR, TREP, C.  sinensis repeat sequence database, TEClass software, Repeatmasker | - |  | EST, RNA-seq, Proteins | - | | Ab initio tools (AUGUSTUS, GlimmerHMM & SNAP), homology search (exonerate, AAT) | 32,579 | BLASTP (SwissProt and TrEMBL), motifs and domains (InterProScan) | (9) | |
| *C. ichangensis* | RepeatModeler, TIGR, TREP, C.  sinensis repeat sequence database, TEClass software, Repeatmasker | - |  | EST, RNA-seq, Proteins | - | | Ab initio tools (AUGUSTUS, GlimmerHMM & SNAP), homology search (exonerate, AAT) | 32,067 | BLASTP (SwissProt and TrEMBL), motifs and domains (InterProScan) | (9) | |
| *Atalantia buxifolia* | RepeatModeler, TIGR, TREP, C.  sinensis repeat sequence database, TEClass software, Repeatmasker | - |  | EST, RNA-seq, Proteins | - | | Ab initio tools (AUGUSTUS, GlimmerHMM & SNAP), homology search (exonerate, AAT) | 28,420 | BLASTP (SwissProt and TrEMBL), motifs and domains (InterProScan) | (9) | |
| *C. unshiu Marc.* | RepeatModeler,  Repeatmasker, RepBase, LTR (LTRharvest, LTRdigest), SSR (mreps) | 39.52 |  | - | - | | MAKER-P | 29,024 | InterProScan, TBLASTX (E-value  _ 1e-20), KEGG database and KAAS | (12) | |
| *C. reticulata* | RepeatModeler, TIGR, TREP, RepeatMasker, HMMsearch  program, | - |  | EST, proteins, RNA-seq | - | | Ab initio tools (AUGUSTUS, GlimmerHMM), homology search (Exonerate, AAT), transcript assembly (Trinity), combined all gene structures (EVM) | 28,820 | - | (13) | |
| *C. reticulata* | EDTA | - |  | - | - | | BRAKER2 | - | KEGG, TrEMBL, and GenBank databases under the E-value 1e-5), GO annotation (InterProScan) | (14) | |
| *Fortunella hindsii* | RepeatModeler, RepBase, RepeatMasker | 43.6 |  | EST, proteins, RNA-seq | Tophat2 | | Ab initio tools (Augustus, GlimmerHMM), homology search (Exonerate, AAT), combined all gene structures (EVM) | 32,257 | BLASTP (E-value < 10e-6) against Swiss-Prot & TrEMBL, motifs and domains (InterProScan) | (15) | |
| *F. hindsii* | EDTA | 46.11 |  | Reference gene models from sweet orange genome, RNA-seq | HISAT2 | | Ab initio tools (Augustus, SNAP), homology search (GeMoMa), transcript-based prediction (Stringtie, GeneMarkS-T, PASA), combined all gene structures (EVM) | 32,563 | NR database, TrEMBL, Pfam, SwissProt, KOG, GO, KEGG | (16) | |
| *Poncirus trifoliata* | RepeatModeler, LTR_finder, LTR_harvest, LTR_retriever, TEClass, RepBase, RepeatMasker | 42.6 |  | EST, proteins, RNA-seq | - | | Ab initio tools (FGENESH+  FGENESH_EST, GenomeScan, PASA assembly  open reading frames (ORFs), and from AUGUSTUS via BRAKER1), homology search (Exonerate) | 25,538 | Pfam | (17) | |
| *P. trifoliata* | RepeatModeler, RepBase, RepeatMasker | 46.5 |  | EST, proteins, RNA-seq | TopHat2, PASA | | Ab initio tools (Augustus, GlimmerHMM), homology search (AAT & Exonerate), combined all gene structures (EVM) | 25,680 | genes were searched against SwissProt and TrEMBL, motifs and domains (InterProScan) | (18) | |
| *P. polyandra* | RepeatModeler, RepeatMasker | 46.0 |  | - | - | | homology-based, de novo, and transcriptome sequencing-based methods, combined all gene structures (EVM) | 20,815 (annotation BUSCO – 96.84%) | BLAST, align protein models in Swiss-Prot, TrEMBL, NCBI NR, GO, COG, KEGG | (19) | |
| *C. limon* | EDTA | 37.05 (primary), 40.93 (alternate) |  | RNA-seq | GSNAP | | Ab initio tools (AUGUSTUS, GeneMarkS-T) | - | eggnog, InterproScan, BLAST + alignment on the Uniprot Uniref100 Viridiplantae  dataset | (20) | |
| *C. limon* | RepeatModeler, RepeatMasker, TEclass | - |  | RNA-seq | HISAT2 | | Ab initio tools (Augustus), homology-based search (Genewise) | 30,528 | BLAST against NR database, Swiss-Prot, KOG, GO database, KEGG pathway analysis | (21) | |
| *C. limon* | RepeatModeler,RepeatScout,PILER,andLTR_FINDER, Uclustsoftware, RepeatMasker | 51.37 |  | - | - | | Ab initio tools (Glim-merHMM, SNAP, GeneID, GENSCAN) | 27,945 | SwissProt, Nr, Pfam, KEGG, InterPro | (22) | |
| *C. australis* | RepeatModeler, RepeatMasker | 54.94 |  | RNA-seq | HISAT2 | | Braker2 | collapsed – 33,873 (nine pseudochromosomes – 31,779)  hap1 – 30,820 (nine pseudochromosomes – 28,725)  hap2 – 29,112 (nine pseudochromosomes – 27,399) | BLASTX (E-value 10e-10) against nr, domains and motifs (InterProScan), GO terms retrieve (InterProScan , Blast2go), KEGG pathway analysis | (23) | |
| Citrus pangenome | RepeatModeler, RepeatMasker, TR_retriever | - |  | RNA-seq, proteins | PASA | | Ab initio tools (AUGUSTUS, GlimmerHMM &  Snap), homology search, combined all gene structures (EVM) | 22,907 to 31,413 | - | (24) | |
| *C. australasica* | RepeatModeler, RepeatMasker | 57.24 |  | RNA-seq | HISAT2 | | Braker3 | collapsed – 41,304 (nine pseudochromosomes – 36,597)  hap1 – 35,305 (nine pseudochromosomes – 30,050)  hap2 – 36,771 (nine pseudochromosomes – 34,139) | BLASTX (E-value 10e-10) against nr, domains and motifs (InterProScan), GO terms retrieve (InterProScan , Blast2go), KEGG pathway analysis | (25) | |
| *C. australasica* | RepeatModeler, RepeatMasker | 55 |  | RNA-seq, proteins | STAR | | BRAKER v2.1.6, GeneMark-EP’s ProtHint pipeline, TSEBRA | 27,358 (primary) 25,461 (alternate) | GO term enrichment (EnTAP, AgriGO) | (26) | |
| *C. australasica* | - | 52.35 |  | RNA-seq, proteins | - | | MAKER | 21,154 | Eggnog-mapper | (27) | |
| *C. inodora* | RepeatModeler, RepeatMasker | 60.82 |  | RNA-seq | HISAT2 | | Braker3 | collapsed – 29,260 (nine pseudochromosomes – 25,862)  hap1 – 30,179 (nine pseudochromosomes – 28,265)  hap2 – 41,852 (nine pseudochromosomes – 30,134) | BLASTX (E-value 10e-10) against nr, domains and motifs (InterProScan), GO terms retrieve (InterProScan , Blast2go), KEGG pathway analysis | (28) | |
| *C. inodora* | RepeatModeler, RepeatMasker | 44 |  | RNA-seq, proteins | STAR | | BRAKER v2.1.6, GeneMark-EP’s ProtHint pipeline, TSEBRA | 28,176 (primary), 27,665 (alternate) | GO term enrichment (EnTAP, AgriGO) | (26) | |
| *C. glauca* | RepeatModeler, RepeatMasker | 55.71 |  | RNA-seq | HISAT2 | | Braker3 | collapsed – 38,490 (nine pseudochromosomes – 30,218)  hap1 – 31,263 (nine pseudochromosomes – 27,722)  hap2 – 33,440 (nine pseudochromosomes – 27,850) | BLASTX (E-value 10e-10) against nr, domains and motifs (InterProScan), GO terms retrieve (InterProScan , Blast2go), KEGG pathway analysis | (28) | |
| *C. glauca* | RepeatModeler, RepeatMasker | 51 |  | RNA-seq, proteins | STAR | | BRAKER v2.1.6, GeneMark-EP’s ProtHint pipeline, TSEBRA | 30,067 (primary), 33,673 (alternate) | GO term enrichment (EnTAP, AgriGO) | (26) | |
| *C. garrawayi* | RepeatModeler, RepeatMasker | 53.2 |  | RNA-seq | HISAT2 | | Braker3 | collapsed – 28,946 (nine pseudochromosomes – 27,548)  hap1 – 27,104 (nine pseudochromosomes – 26,553)  hap2 – 34,366 (nine pseudochromosomes – 27,020) | BLASTX (E-value 10e-10) against nr, domains and motifs (InterProScan), GO terms retrieve (InterProScan , Blast2go), KEGG pathway analysis | (28) | |
| *C. changshanensis* | MITE repeats (MITE-Hunter), LTR repeats (LTR-Retriver), RepeatModeler, RepeatMasker | 52.17 (primary), 50.24 (hap1), 48.54 (hap2) |  | RNA-seq, proteins | HISAT2 | | BRAKE v2.1.4, Maker | 29,775 (primary), 29,716 (hap1), 29,806 (hap2) | BLASTP (E-value < 10e-4) against citrus plant protein databases, other plant protein databases, Swiss-Prot, domains and GO terms (nterProScan) | (29) | |
| Citrus pangenome | RepeatMasker, CENSOR, BLASTER, TRF, Mreps, RMSSR | 42.5 - 47.3 |  | RNA-seq, proteins, ESTs | HISAT2 | | EuGene Eukaryotic Pipeline | 28,090 to 30,101 (93.1% - 94.8% BUSCO) | BLAST against Swiss-Prot and TrEMBL databases, InterProScan, KEGG against KOfam database using KofamScan, RRGPredictor to classify proteins into classes | (30) | |

EST – Expressed sequence tags, RNA-seq – RNA sequencing, RNA-PET – RNA paired-end tag sequencing, EDTA - Extensive de-novo TE Annotator, TRF – Tandem repeat finder, NR – Non-redundant, SNAP - Semi-HMM-based Nucleic Acid Parser, EVM – EvidenceModeler, GeMoMa – Gene model mapper, BLAST - Basic Local Alignment Search Tool, TE – Transposable elements, PASA - Program to Assemble Spliced Alignments, alt-EST – alternative EST, KEGG - Kyoto Encyclopedia of Genes and Genomes, KAAS - KEGG Automatic Annotation Server, GO – Gene ontology, COG (NCBI Clusters of Orthologous Groups of Proteins, KOfam - hidden Markov model), KOG - EuKaryotic Orthologous Groups

**References**

1. Xu Q, Chen L-L, Ruan X, Chen D, Zhu A, Chen C, et al. The draft genome of sweet orange (Citrus sinensis). Nat Genet. 2013;45(1):59-66.

2. Wang L, Huang Y, Liu Z, He J, Jiang X, He F, et al. Somatic variations led to the selection of acidic and acidless orange cultivars. Nature Plants. 2021;7(7):954-65.

3. Wu B, Yu Q, Deng Z, Duan Y, Luo F, Gmitter Jr F. A chromosome-level phased genome enabling allele-level studies in sweet orange: a case study on citrus Huanglongbing tolerance. Horticulture Research. 2023;10(1):uhac247.

4. Xiong Z, Yin H, Wang N, Han G, Gao Y. Chromosome-level genome assembly of navel orange cv. Gannanzao (Citrus sinensis Osbeck cv. Gannanzao). G3 Genes|Genomes|Genetics. 2023.

5. Wang N, Chen P, Xu Y, Guo L, Li X, Yi H, et al. Phased genomics reveals hidden somatic mutations and provides insight into fruit development in sweet orange. Horticulture Research. 2023:uhad268.

6. Gao Y, Xu J, Li Z, Zhang Y, Riera N, Xiong Z, et al. Citrus genomic resources unravel putative genetic determinants of Huanglongbing pathogenicity. Iscience. 2023;26(2).

7. Wu GA, Prochnik S, Jenkins J, Salse J, Hellsten U, Murat F, et al. Sequencing of diverse mandarin, pummelo and orange genomes reveals complex history of admixture during citrus domestication. Nat Biotechnol. 2014;32(7):656-62.

8. Zhang Y, Barthe G, Grosser JW, Wang N. Transcriptome analysis of root response to citrus blight based on the newly assembled Swingle citrumelo draft genome. BMC Genomics. 2016;17(1):1-10.

9. Wang X, Xu Y, Zhang S, Cao L, Huang Y, Cheng J, et al. Genomic analyses of primitive, wild and cultivated citrus provide insights into asexual reproduction. Nat Genet. 2017;49(5):765-72.

10. Lu Z, Huang Y, Mao S, Wu F, Liu Y, Mao X, et al. The high-quality genome of pummelo provides insights into the tissue-specific regulation of citric acid and anthocyanin during domestication. Horticulture Research. 2022;9:uhac175.

11. Zheng W, Zhang W, Liu D, Yin M, Wang X, Wang S, et al. Evolution‐guided multiomics provide insights into the strengthening of bioactive flavone biosynthesis in medicinal pummelo. Plant Biotechnol J. 2023.

12. Shimizu T, Tanizawa Y, Mochizuki T, Nagasaki H, Yoshioka T, Toyoda A, et al. Draft sequencing of the heterozygous diploid genome of Satsuma (Citrus unshiu Marc.) using a hybrid assembly approach. Frontiers in genetics. 2017;8:180.

13. Wang L, He F, Huang Y, He J, Yang S, Zeng J, et al. Genome of wild mandarin and domestication history of mandarin. Molecular plant. 2018;11(8):1024-37.

14. Zhu C, You C, Wu P, Huang Y, Zhang R, Fan Z, et al. The gap-free genome and multi-omics analysis of Citrus reticulata ‘Chachi’reveal the dynamics of fruit flavonoid biosynthesis. Horticulture Research. 2024;11(8):uhae177.

15. Zhu C, Zheng X, Huang Y, Ye J, Chen P, Zhang C, et al. Genome sequencing and CRISPR/Cas9 gene editing of an early flowering Mini‐Citrus (Fortunella hindsii). Plant Biotechnol J. 2019;17(11):2199-210.

16. Wang N, Song X, Ye J, Zhang S, Cao Z, Zhu C, et al. Structural variation and parallel evolution of apomixis in citrus during domestication and diversification. National Science Review. 2022;9(10):p.nwac114.

17. Peng Z, Bredeson JV, Wu GA, Shu S, Rawat N, Du D, et al. A chromosome‐scale reference genome of trifoliate orange (Poncirus trifoliata) provides insights into disease resistance, cold tolerance and genome evolution in Citrus. The Plant Journal. 2020;104(5):1215-32.

18. Huang Y, Xu Y, Jiang X, Yu H, Jia H, Tan C, et al. Genome of a citrus rootstock and global DNA demethylation caused by heterografting. Horticulture research. 2021;8(1):1-13.

19. Zhang S, Chen J, Zhang C, Zhang S, Zhang X, Gao L, et al. Insights into identifying resistance genes for cold and disease stresses through chromosome-level reference genome analyses of Poncirus polyandra. Genomics. 2023;115(3):110617.

20. Guardo MD, Moretto M, Moser M, Catalano C, Troggio M, Deng Z, et al. The haplotype-resolved reference genome of lemon (Citrus limon L. Burm f.). Tree Genet Genom. 2021;17(6):1-12.

21. Bao Y, Zeng Z, Yao W, Chen X, Jiang M, Sehrish A, et al. A gap-free and haplotype-resolved lemon genome provides insights into flavor synthesis and huanglongbing (HLB) tolerance. Horticulture Research. 2023;10(4):uhad020.

22. Yu H, Zhang C, Lu C, Wang Y, Ge C, Huang G, et al. The lemon genome and DNA methylome unveil epigenetic regulation of citric acid biosynthesis during fruit development. Horticulture Research. 2024;11(3):uhae005.

23. Nakandala U, Masouleh AK, Smith MW, Furtado A, Mason P, Constantin L, et al. Haplotype resolved chromosome level genome assembly of Citrus australis reveals disease resistance and other citrus specific genes. Horticulture Research. 2023;10(5):uhad058.

24. Huang Y, He J, Xu Y, Zheng W, Wang S, Chen P, et al. Pangenome analysis provides insight into the evolution of the orange subfamily and a key gene for citric acid accumulation in citrus fruits. Nat Genet. 2023:1-12.

25. Nakandala U, Furtado A, Masouleh AK, Smith MW, Williams DC, Henry RJ. The genome of Citrus australasica reveals disease resistance and other species specific genes. BMC Plant Biol. 2024;24(1):260.

26. Singh K, Huff M, Liu J, Park J-W, Rickman T, Keremane M, et al. Chromosome-Scale, De Novo, Phased Genome Assemblies of Three Australian Limes: Citrus australasica, C. inodora, and C. glauca. Plants. 2024;13(11):1460.

27. Tian Y, Liang T, Peng H, Wang Q, Luo X, Xu R, et al. Chromosome-scale genome assembly provides insights into the evolution and color synthesis of finger lemon (Citrus australasica). Tropical Plants. 2024;3(1).

28. Nakandala U, Furtado A, Masouleh AK, Smith MW, Mason P, Williams DC, et al. The genomes of Australian wild limes. Plant Mol Biol. 2024;114(5):102.

29. Miao C, Wu Y, Wang L, Zhao S, Grierson D, Xu C, et al. Haplotype-resolved chromosome-level genome assembly of Huyou (Citrus changshanensis). Scientific Data. 2024;11(1):605.

30. Droc G, Giraud D, Belser C, Labadie K, Duprat S, Cruaud C, et al. A super-pangenome for cultivated citrus reveals evolutive features during the allopatric phase of their reticulate evolution. bioRxiv. 2024:2024.10. 17.618847.
