# Supplementary material for: Citrus genomes: past, present and future
Source: Hortic Res. 2025 Feb 4;12(5):uhaf033. doi: 10.1093/hr/uhaf033 (PMC11992330; doi:10.1093/hr/uhaf033)
Supplement: Web_Material_uhaf033 [file web_material_uhaf033.zip › Supplemnetary Table S3.docx]

| **Species** | **Difference in size (Mb)** | **Difference in gene number** | **Sequence variations** | **Reference** |
| --- | --- | --- | --- | --- |
| *C. sinensis* | - | 131 | SNVs, insertions, deletions, tandem duplications | (1) |
|  | 15.2 | 995 | SNVs, insertions, deletions | (2) |
|  | - | 92 | - | (3) |
| *C. limon* | 11.9 | - | The number and the distributions of the TEs | (4) |
|  | 34.2 | - | - | (5) |
| *C. australis* | 7.8 (9chr) | 70 (9chr) | translocations, duplications | (6) |
| *C. australasica* | 6 (9chr) | 4,089 (9chr) | protein coding genes, inversions, translocations, duplications | (7) |
|  | 1.5 | 1,897 | SNVs, inversions, translocations, duplications, insertions, deletions, HDR | (8) |
| *C. inodora* | 3.4 (9chr) | 1,869 (9chr) | translocations or duplications | (9) |
|  | 4.9 | 511 | SNVs, inversions, translocations, duplications, insertions, deletions, HDR | (8) |
| *C. glauca* | 11.6 (9chr) | 128 (9chr) | translocations, duplications | (9) |
|  | 2.8 | 3,606 | SNVs, inversions, translocations, duplications, insertions, deletions, HDR | (8) |
| *C. garrawayi* | 1.3 (9chr) | 467 (9chr) | Inversions, translocations, duplications | (9) |
| *C. changshanensis* | 11.6 | 90 | The number of TEs, inversions, translocations, duplications | (10) |

**Supplementary Table S3** Structural variations between haplotype assemblies in terms of the size, gene number and other sequence variations

SNVs – Single nucleotide variants, TEs – Transposable elements, highly diverged regions

**References**

1. Wu B, Yu Q, Deng Z, Duan Y, Luo F, Gmitter Jr F. A chromosome-level phased genome enabling allele-level studies in sweet orange: a case study on citrus Huanglongbing tolerance. Horticulture Research. 2023;10(1):uhac247.

2. Wang N, Chen P, Xu Y, Guo L, Li X, Yi H, et al. Phased genomics reveals hidden somatic mutations and provides insight into fruit development in sweet orange. Horticulture Research. 2023:uhad268.

3. Gao Y, Xu J, Li Z, Zhang Y, Riera N, Xiong Z, et al. Citrus genomic resources unravel putative genetic determinants of Huanglongbing pathogenicity. Iscience. 2023;26(2).

4. Guardo MD, Moretto M, Moser M, Catalano C, Troggio M, Deng Z, et al. The haplotype-resolved reference genome of lemon (Citrus limon L. Burm f.). Tree Genet Genom. 2021;17(6):1-12.

5. Bao Y, Zeng Z, Yao W, Chen X, Jiang M, Sehrish A, et al. A gap-free and haplotype-resolved lemon genome provides insights into flavor synthesis and huanglongbing (HLB) tolerance. Horticulture Research. 2023;10(4):uhad020.

6. Nakandala U, Masouleh AK, Smith MW, Furtado A, Mason P, Constantin L, et al. Haplotype resolved chromosome level genome assembly of Citrus australis reveals disease resistance and other citrus specific genes. Horticulture Research. 2023;10(5):uhad058.

7. Nakandala U, Furtado A, Masouleh AK, Smith MW, Williams DC, Henry RJ. The genome of Citrus australasica reveals disease resistance and other species specific genes. BMC Plant Biol. 2024;24(1):260.

8. Singh K, Huff M, Liu J, Park J-W, Rickman T, Keremane M, et al. Chromosome-Scale, De Novo, Phased Genome Assemblies of Three Australian Limes: Citrus australasica, C. inodora, and C. glauca. Plants. 2024;13(11):1460.

9. Nakandala U, Furtado A, Masouleh AK, Smith MW, Mason P, Williams DC, et al. The genomes of Australian wild limes. Plant Mol Biol. 2024;114(5):102.

10. Miao C, Wu Y, Wang L, Zhao S, Grierson D, Xu C, et al. Haplotype-resolved chromosome-level genome assembly of Huyou (Citrus changshanensis). Scientific Data. 2024;11(1):605.
